# Life-history characteristics and historical factors are important to explain regional variation in reproductive traits and genetic diversity in perennial mosses

**DOI:** 10.1093/aob/mcad045

**Published:** 2023-03-16

**Authors:** Irene Bisang, Johan Ehrlén, Lars Hedenäs

**Affiliations:** Department of Botany, Swedish Museum of Natural History, Box 50007, SE-104 05 Stockholm, Sweden; Department of Ecology, Environment and Plant Sciences, Stockholm University, SE-106 91 Stockholm, Sweden; Department of Botany, Swedish Museum of Natural History, Box 50007, SE-104 05 Stockholm, Sweden

**Keywords:** Bryophytes, *Drepanocladus trifarius*, *Drepanocladus turgescens*, intraspecific genetic differentiation, molecular sex identification, perennial plant, regional variation, reproductive traits, sex expression, sex ratio, sporophyte production

## Abstract

**Background and Aims:**

Plants have evolved an unrivalled diversity of reproductive strategies, including variation in the degree of sexual vs. clonal reproduction. This variation has important effects on the dynamics and genetic structure of populations. We examined the association between large-scale variation in reproductive patterns and intraspecific genetic diversity in two moss species where sex is manifested in the dominant haploid generation and sex expression is irregular. We predicted that in regions with more frequent realized sexual reproduction, populations should display less skewed sex ratios, should more often express sex and should have higher genetic diversity than in regions with largely clonal reproduction.

**Methods:**

We assessed reproductive status and phenotypic sex in the dioicous long-lived *Drepanocladus trifarius* and *D. turgescens*, in 248 and 438 samples across two regions in Scandinavia with frequent or rare realized sexual reproduction, respectively. In subsets of the samples, we analysed genetic diversity using nuclear and plastid sequence information and identified sex with a sex-specific molecular marker in non-reproductive samples.

**Key Results:**

Contrary to our predictions, sex ratios did not differ between regions; genetic diversity did not differ in *D. trifarius* and it was higher in the region with rare sexual reproduction in *D. turgescens*. Supporting our predictions, relatively more samples expressed sex in *D. trifarius* in the region with frequent sexual reproduction. Overall, samples were mostly female. The degree of sex expression and genetic diversity differed between sexes.

**Conclusions:**

Sex expression levels, regional sex ratios and genetic diversity were not directly associated with the regional frequency of realized sexual reproduction, and relationships and variation patterns differed between species. We conclude that a combination of species-specific life histories, such as longevity, overall degree of successful sexual reproduction and recruitment, and historical factors are important to explain this variation. Our data on haploid-dominated plants significantly complement plant reproductive biology.

## INTRODUCTION

Plants have evolved an unrivalled diversity of sexual systems and structural, functional and temporal variation in the display of reproductive organs that influence mating success (Barrett, 2002; Jesson and Garnock-Jones, 2012). This diversification in plants is largely considered to have evolved to increase control over mating, given their immobility and dependence on water, wind or animals for dispersal of male gametes (Barrett and Harder, 2017). Much of our knowledge of plant reproductive systems is based on studies with flowering plants, in which sporophytes are the independent life cycle phase. However, plants with gametophytes as dominant life stages exhibit fundamental differences in characteristics that affect mating processes (e.g. Pannell, 2017). These include that sex is manifested in the haploid gametophyte generation, resource allocation to sexes occurs primarily in the gametophyte generation, and there is a possibility of intragametophytic selfing in gametophytes with both sexes, leading to completely homozygous sporophytes and genetically identical spores (Renner, 2014; Haig, 2016; Renner *et al.*, 2017). Furthermore, sex determination of unisexual gametophytes occurs at meiosis when sex-determining loci segregate, rather than at syngamy (Haig, 2016; Carey *et al.*, 2021). The dioecious mating system has originated independently in many different plant lineages (McDaniel *et al.*, 2013; Villarreal and Renner, 2013; Renner, 2014). Dioecy offers benefits related to genetic diversity in offspring, sexual specialization or escape from inbreeding depression, but it also entails that only half of the individuals produce progeny and bears the risk of not achieving fertilization because of the immobility of plants (Barrett, 2002; Barrett and Harder, 2017; Käfer *et al.*, 2017). The latter can be accentuated when the two sexes are spatially segregated (Bierzychudek and Eckhart, 1988). Given these limitations of sexual reproduction, reproduction by asexual (vegetative) means can be an alternative (Meloni *et al.*, 2013; Barrett, 2015). Clonal growth (i.e. the multiplication of vegetative parts) or the production of specialized vegetative diaspores, and various combinations thereof and with sexual reproduction, are widespread among land plants (Vallejo-Marín *et al.*, 2010; Frey and Kürschner, 2011; Barrett, 2015).

Different mating systems and modes of reproduction affect the dynamics and genetic structure of populations and have important ecological and evolutionary consequences (Charpentier, 2002; Fritz, 2009; Eckert *et al.*, 2016; Liu *et al.*, 2016). For example, in some plant lineages, separate sex is correlated with asexual reproduction (‘reproductive assurance’; Longton and Schuster, 1983; During, 2007; Field *et al.*, 2013*a*), but a general association has been questioned (Crawford *et al.*, 2009; Laenen *et al.*, 2015). Clonal reproduction was reported to preserve genetic diversity and to reinforce spatial genetic structure in seed plants (e.g. Wang *et al.*, 2004; Dering *et al.*, 2016). Dioecious clonal species of flowering plants have been found to exhibit greater sex ratio heterogeneity, stronger sex ratio bias and larger spatial sex segregation than non-clonal species (Field *et al.*, 2013*b*). Female and male plants can differ in life-history traits, including clonal growth capacity or mortality as an effect of sex-differential reproductive costs that can result in spatial segregation of the sexes, occurrence in different microhabitats and sex-specific physiology (Zhang *et al.*, 2014; and references therein). The time since plants colonized a new locality is expected to reinforce differences between sexes based on sex-specific performance. Different historical factors, such as founder events, glaciation history, climate fluctuation or immigration routes, have indeed been shown to influence regional variation in reproductive traits at various scales (e.g. Alsos *et al.*, 2005; Field *et al.*, 2013*b*; Hedenäs, 2017; Carter, 2021; Boquete *et al.*, 2023). Finally, such differences are likely to be reflected in sex-related patterns of genetic diversity and genetic structure (Dering *et al.*, 2016; Zhai *et al.*, 2016).

In this study, we investigate two moss species, representing bryophytes, non-vascular plants with a life cycle dominated by the free-living multicellular haploid gametophyte (Haig, 2016). At least 60 % of all extant bryophytes possess sexually specialized male or female gametophytes (McDaniel and Perroud, 2012; Villarreal and Renner, 2013), i.e. they are dioicous (for terminology of dioecy vs. dioicy, see Wyatt, 1985). Sexual reproduction and spore production are geographically and temporally restricted in many dioicous bryophytes (Bisang and Hedenäs, 2005, 2008; Hedenäs and Bisang, 2019*a*). This can be the result of no sexual organs being produced (i.e. plants remain non-reproductive) or of fertilization failure (Supplementary data Table S1; Longton and Schuster, 1983; Bisang and Hedenäs, 2005). Successful fertilization, eventually resulting in a spore-producing sporophyte, can be hampered by lack of water (Haig, 2016) or other unsuitable environmental conditions, or by spatial segregation of the sexes and skewed sex ratios (Sundberg, 2002; Bisang *et al.*, 2004; Haig and Wilczek, 2006). Indeed, many populations, or even species, rely entirely on asexual reproduction, i.e. clonal proliferation without or with specialized vegetative propagules (Frey and Kürschner, 2011). Recent studies with bryophyte species have demonstrated distinct intraspecific genetic diversity (e.g. Désamoré *et al.*, 2016; Hedenäs, 2019), and some studies have suggested that long-lived species with rare sporophyte production exhibit low genetic diversity (Fritz, 2009). Cronberg (2002) postulated that sporophyte frequency might serve as an indicator of population genetic variability. Nevertheless, the exact relationship between this variation and reproductive mode (i.e. levels of sexual reproduction vs. clonality) within and across bryophyte species, and whether genetic diversity is sex specific, remain poorly explored.

We examined large-scale geographical variation in reproductive patterns and their association with intraspecific genetic diversity and differentiation in the two perennial dioicous wetland mosses *Drepanocladus trifarius* (F.Weber & D.Mohr) Broth. ex Paris and *D. turgescens* (T.Jensen) Broth. For each species, we sampled individual moss patches in two geographically separated regions that differed in the frequency at which each species reproduces sexually, and asked the following questions. First, do sex ratios differ between regions with regular successful sexual reproduction (sporophyte production) and regions where the species is largely clonal? Second, does reproductive state, appraised as the proportion of samples with sexual structures (sex expression), differ between regions? We predicted that in regions with successful sexual reproduction, sex ratios would be less strongly skewed, and sex expression would be more common, because relatively balanced sex ratios increase the chance, and the formation of reproductive organs is a prerequisite, for successful fertilization. Third, do genetic diversity and composition differ between samples in regions with regular and regions with rare or no realized sexual reproduction? We predicted that genetic diversity would be higher in regions where sexual reproduction is more frequent than in regions where clonal growth dominates, because of relaxed mate limitations, enhanced fertilization success and higher genetic mixing. Fourth, does genetic diversity and its regional variation have a sex-specific component? We compared genetic diversity and composition between males and females within and between regions and predicted that the rare sex would exhibit lower genetic diversity.

## MATERIALS AND METHODS

### Study species, their life cycles and sex ratio terminology

The two study species are dioicous pleurocarpous wetland mosses of the family Amblystegiaceae that differ in ecological niche, distribution pattern (Hedenäs, 2003; Hedenäs *et al.*, 2003; Hedenäs and Bisang, 2012) and reproductive performance. Mosses are one of three lineages that constitute the monophyletic bryophytes (Su *et al.*, 2021), which share a haploid-dominant life cycle (for details on the bryophyte life cycle and bryophyte-specific terms, see Supplementary data Table S1). In this paper, we term individuals, patches and populations without sexual organs (i.e. without sex expression) ‘non-reproductive’ (Bisang *et al.*, 2020). We distinguish between phenotypic sex ratios (based on reproductive populations) and sex ratios of non-reproductive plants identified with molecular methods (Bisang *et al.*, 2020: fig. 1).

**Fig. 1.**
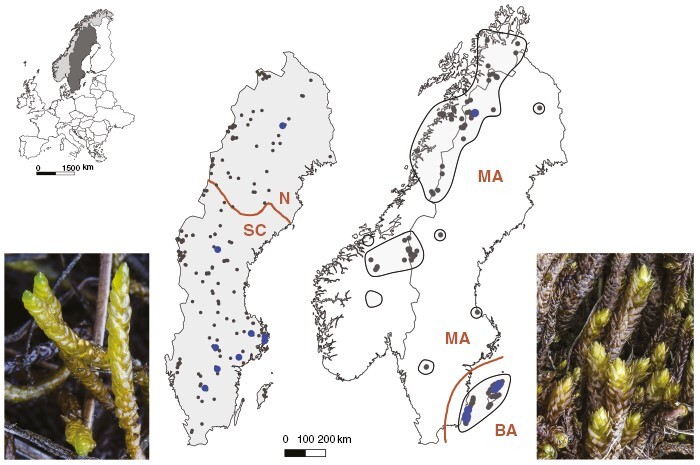
Study area and study species. Inset, map of Europe, with Norway shaded in light grey and Sweden in dark grey. For each species, we compared two study regions with different levels of realized sexual reproduction, estimated based on sporophyte frequencies (blue dots: one or several sporophyte occurrences). Left, *Drepanocladus trifarius*, study regions: N, north Sweden; SC, south-central Sweden. Right, *Drepanocladus turgescens*, study regions: BA, Baltic Sea islands Gotland and Öland; MA, mainland Scandinavia. Shading, distribution of the target species in the study regions; grey dots, sample locations. For details, see the Supplementary data (Figs S1 and S2; Table S2). Photographs: Lars Hedenäs.

*Drepanocladus trifarius* is relatively common in the northern temperate to arctic zones, including in the study area (Fig. 1; Supplementary data Fig. S1). Typically, it grows in constantly wet habitats. Across its European distribution, less than one-third of local occurrences of the species were expressing sex (30 %), only 15 % of reproductive female occurrences formed sporophytes, and sporophyte formation varied between regions (Bisang *et al.*, 2014). Across Europe, at both phenotypic and genotypic levels and at different spatial scales, there were two to three times as many female plants or populations as males (Bisang *et al.*, 2006, 2015; Hedenäs *et al.*, 2010).

*Drepanocladus turgescens* is frequent in the Scandinavian mountain range, scattered in the lowlands, and regionally abundant on the Baltic Sea islands Gotland and Öland (Fig. 1), whereas its distribution is fragmented and it has declined strongly in Central Europe owing to habitat loss (Hedenäs, 2014; Hedenäs and Bisang, 2019*b*). It grows on calcareous substrates in periodically wet depressions or sites with trickling water. In the core distribution area of the species across the Nordic and Baltic countries, only 20 % of the surveyed samples were expressing sex, 22 % of reproductive female samples carried sporophytes (Bisang *et al.*, 2014), and sporophyte formation was regionally restricted and episodic (Fig. 1; Supplementary data Fig. S2). Overall phenotypic and genotypic sex ratios in Scandinavia were strongly female-skewed, with two to three female samples per male sample (Bisang *et al.*, 2014; Hedenäs *et al.*, 2016; Hedenäs and Bisang, 2019*a*; unpubl. data, this study).

### Study regions

The study area is situated in the Scandinavian countries Sweden and Norway, extending from 56.24°N (southern end of the Baltic Sea island Öland) to 69.35°N (northern Norway, Troms) (Fig. 1). It covers a bioclimatic gradient from the warm-temperate (nemoral), humid with warm summers climate zone in the south and along the western coasts to the dominating cold-temperate (snow), humid with cool summers climate zone, which includes a polar tundra climate in the mountain range above the tree line (following Köppen-Geiger climate classification: Dierssen, 1996; Kottek *et al.*, 2006). From the west coast to the east, it encompasses a gradient of increasing continentality from 9.54 to 22.06°E (Dierssen, 1996; Kottek *et al.*, 2006). Within this area, we selected, for each of the species, two study regions that differed in the degree of sexual reproduction estimated by sporophyte frequencies (Fig. 1). Sporophyte frequency estimates were based on expert knowledge after 40 years of fieldwork by the senior author, herbarium records and literature.

For *D. trifarius*, we compared reproductive performance between two regions in Sweden, here termed north and south-central (Fig. 1). We used phytogeographical zones rather than latitude to delimit study regions with different sexual reproduction levels. Phytogeographical zones are viewed as a proxy for environmental conditions; they are largely based on the vegetation structure and composition and also consider elevation, hence they do not follow strictly latitudinal gradients (Sjörs, 1999). The south-central region is located in the phytogeographical boreo-nemoral zone and southern boreal subzones, and the northern region extends over the middle and northern boreal subzones, including part of the mountain range (Sjörs, 1999). In northern Sweden, sporophytes have been observed in only two localities, whereas they occur more regularly further south in Scandinavia (Fig. 1; Supplementary data Fig. S1; Bisang *et al.*, 2006, 2014).

For *D. turgescens*, we compared reproductive performance between mainland Scandinavia and the islands Gotland and Öland in the Baltic Sea (Fig. 1; Supplementary data Fig. S2). The climate and environment on these islands are influenced by the surrounding sea, limestone bedrock and generally shallow soils. We observed ample sporophytes during several seasons on Gotland and Öland (Hedenäs and Bisang, 2019*a*). In contrast, *D. turgescens* rarely produces sporophytes on mainland Scandinavia (Bisang *et al.*, 2014).

### Sampling design and data collection

Reproductive trait data were largely sampled using herbarium collections from Sweden and Norway stored at the Swedish Museum of Natural History (S) collected up to March 2018. From the entire holdings of the study species at S up to this date, we discarded duplicate specimens, multiple collections from the same locality and specimens containing only a few shoots. We did not consider other features of the specimens (e.g. specimen size or label information) in the sample selection. We aimed at an even geographical distribution and a balanced study intensity that reflected the regional density of occurrences. To reduce problems associated with an uneven distribution of available collections, we carried out additional field collecting in 2018 and 2019 and incorporated vouchers for these collections also. Each specimen eventually studied thus originated from an individual patch of the respective species, termed ‘sample’ hereafter. Sample locations were separated by ≥100 m to avoid sampling from the same clone (Bisang, *et al.*, 2015). In their natural environment, both study species usually form patches that can cover ≤0.5 m^2^, but are usually smaller. A herbarium collection is typically up to ~0.5 dm^2^. In a patch, both sexes can occur within distances of a few centimetres (Bisang, *et al.*, 2015), but because of the ability of the species to expand clonally, many patches are likely to be unisexual. For *D. trifarius*, we showed that it is more likely than by chance to encounter the same sex within 25 cm of a patch (Bisang, *et al.*, 2015). In agreement, <5 % of the 84 reproductive samples of *D. trifarius* and of the 101 reproductive samples of *D. turgescens* contained both male and female sexual structures (Supplementary data Table S2). For genetic diversity analyses and genetic sex identification, we randomly picked one individual shoot per sample to represent the patch (see below, B).

Sexual branches and sporophytes in the study species persist for at least two reproductive cycles in the field and remain in dried specimens, once collected. The large number of collections investigated in this study provides a reasonable estimate of reproductive patterns across a region. Natural History collections offer major advantages for trait, genetic and other types of analyses, such as their extensive geographical coverage, which can be accesses in limited time (e.g. Heberling *et al.*, 2019). At the same time, they are associated with some potential biases (Bisang *et al.*, 2014; Daru *et al.*, 2018). Sporophytes are recognizable during field collection, and sporophytic plants might be collected preferably in species with rare sexual reproduction. However, this should be less of a problem when, for example, comparing relative sporophyte frequency in different areas, because there is no obvious reason why such a potential bias should depend on geographical location. Given the limited travel distances of sperm (Bisang *et al.*, 2004), the occurrence of sporophytes indicates a higher probability of that both male and female sexual branches are present in a patch. Sexual branches are not easily observed in the field, and sporophytic samples are likely more often to include the rarer sex than samples without sporophytes. Excluding sporophytic collections, however, entails that sex expression and samples of the rarer sex might be underestimated. The true sex expression rates in a natural population are likely to fall in between these two estimates. We chose to exclude specimens with sporophytes for analyses of sex expression levels and sex ratios. This was because we judged that the risk of underestimating the frequency of the rare sex and sex expression was small and independent of region using this approach, because we studied species with overall infrequent sporophyte production. Furthermore, using Natural History collections covering long temporal spans can be problematic if trait distributions in the field are not stable over time. However, for our study this should constitute a problem only if the distribution of collection years differs between the areas being compared. We tested whether collection year had an effect on the reproductive parameters of interest. This was the case only for the proportion of samples with sexual structures (‘reproductive state’) in *D. turgescens*. However, given that the distribution of collection years did not differ between the study regions (generalized linear model, normal distribution, identity function; *n* = 411, *P* = 0.356), we judged that this should not constitute a problem for our study purposes.

### Reproductive traits

To study reproductive traits of *D. trifarius*, we picked a total of 248 samples from Sweden: 83 from the northern region and 165 from the south-central region. Ten samples bore sporophytes (one north; nine south-central; Supplementary data Table S2; Fig. S1A). The reproductive subset out of the total of 248 samples included those 84 samples that carried sexual structures but no sporophytes (18 north and 66 south-central). Four of the 84 samples had both male and female sexual organs. We used this subset to assess the phenotypic sex ratio. Of the154 non-reproductive samples, 64 originated from the north and 90 from the south-central region.

For *D. turgescens*, we had 438 samples accessible from the two study regions, mainland Scandinavia and Baltic Sea islands (184 mainland and 254 Baltic). Twenty-four samples contained sporophytes (one mainland and 23 Baltic; Supplementary data Table S2; Fig. S2A). The reproductive subset out of the total of 438 samples included those 101 samples that bore sexual structures but not sporophytes (48 mainland and 53 Baltic Sea islands). Five of these 101 samples contained both male and female reproductive organs (two mainland and three Baltic). We used the reproductive subset (101) to assess the phenotypic sex ratio. Furthermore, to analyse the sex ratio in non-reproductive samples, we picked a subset of similar size (102) out of the 313 non-reproductive samples, aiming at a roughly even geographical distribution across each study region (52 mainland and 50 Baltic Sea islands).

We examined each sample carefully under a dissecting scope for female sexual branches (perichaetia) without sporophytes, male sexual branches (perigonia) and sporophytes, for 20 min or until we observed reproductive structures. We classified each sample based on its reproductive state as M (male), F (female), F + M (male and female), sporophytic (implying the presence of both F and M) or non-reproductive (NR).

### Molecular data and molecular sex identification

We analysed intraspecific genetic diversity and genetic composition between regions with different levels of sporophyte production and between sexes in both species based on sequence information of the nuclear internal transcribed spacers 1 and 2 (ITS), glyceraldehyde 3-phosphate dehydrogenase (*gpd*) and the plastid *rpl*16 G2 intron (*rpl*16) in one randomly picked individual shoot per sample. We complemented the samples in the datasets for trait analyses described above with additional samples from previous studies.

For *D. trifarius*, we re-analysed 86 samples from a previous study (Hedenäs, 2019) to estimate genetic diversity in the two regions (Dataset GenVar: 42 north and 44 south-central; Supplementary data Table S2). Sixty-seven of these 86 previously analysed samples were also included in the trait analyses in this study (Supplementary data Fig. S1B). We refer to the paper by Hedenäs (2019) for DNA extraction, PCR, sequencing and GenBank numbers. These 86 samples included both reproductive and non-reproductive samples, and the latter had been sexed with a female-targeting molecular marker following Korpelainen *et al.* (2008) (dataset GenSex: 41 female and 45 male; Supplementary data Table S2). We compared genetic diversity between female and male samples within each region and across regions, respectively.

In *D. turgescens*, we estimated genetic diversity in the two regions by analysing a subset of 78 samples from the reproductive trait dataset plus ten additional samples from the same regions from the study by Hedenäs (2014) (88 in total; dataset GenVar: 33 Baltic Sea islands and 55 mainland samples; Supplementary data Table S2; Fig. S2C). For 63 of these 88 samples, sequence data were available from the study by Hedenäs (2014), and for the remaining 15 samples, we generated new sequences of the three markers following Hedenäs (2014) for DNA extraction, PCR and sequencing. We edited and assembled nucleotide sequence fragments for each DNA region using PhyDE® v.0.9971 (http://www.phyde.de/index.html; accessed 2 March 2021). We aligned the assembled sequences manually in PhyDE®. Regions of partly incomplete data at the beginning and end of the sequences were excluded from subsequent analyses. We included gap information in the analyses after using the simple indel coding of Simmons and Ochoterena (2000) in SeqState (Müller, 2005). The sequence alignments used in the analyses are available on request. GenBank accession numbers are provided in the Supplementary data (Table S3). Although ITS paralogues are occasionally encountered in bryophytes (e.g. Košnar *et al.*, 2012; Hedenäs *et al.*, 2019), ITS chromatograms included in the present study did not show ‘messy’ patterns or noise that could suggest paralogy, and the 5.8S gene was invariable among all samples (Shaw *et al.*, 2002; Feliner and Rosselló, 2007). Therefore, the revealed ITS variation was interpreted as being among homologous haplotypes. We identified sex in the selected 102 non-reproductive samples using the molecular method described by Hedenäs *et al.* (2016) (dataset NR; Supplementary data Table S2). We compared genetic diversity between female and male samples within each region and across regions for a subset of the sexed or reproductive specimens out of R and NR (Dataset GenSex: Baltic Sea islands, 15F and 16M; mainland, 22F and 15M; Supplementary data Table S2).

### Data analyses of reproductive traits

For each species and region, we calculated the sex ratio as the proportion of male and female samples that were male [M/(M + F)]. For *D. trifarius*, we determined the phenotypic sex ratio for the non-sporophytic reproductive samples (*n* = 84). The four samples containing male and female reproductive organs were counted as both M and F, which resulted in ‘*n*’ for the phenotypic sex ratio of 88. We did not assess the sex ratio in non-reproductive plants, because the sex-identified samples from the study by Hedenäs (2019) were not picked at random with respect to sex. For *D. turgescens*, we computed sex ratios for both reproductive (*n* = 101; five with male and female structures, thus *n* = 106 for phenotypic sex ratio) and non-reproductive samples identified by a molecular marker (*n* = 102) for each region. We tested whether sex ratios differed from an expected unbiased sex ratio (0.5) with Pearson’s χ^2^ tests. For each species and region, we quantified sex expression as the proportion of all samples that were reproductive, i.e. bearing male or female reproductive structures but no sporophytes (*D. trifarius*, *n* = 238; *D. turgescens*, *n* = 414). For calculation of the proportion of male- and female-expressing samples, we quantified males and females each with a value of 0.5 in the samples with both sexes.

For *D. trifarius*, we tested for regional differences (north vs. south-central, fixed factor) in sex (male vs. female) and reproductive state (sex expressing or not, excluding sporophytic samples), using generalized linear models with a binomial distribution and a log-link function. For *D. turgescens*, we applied a similar model to test for regional differences (Baltic Sea islands vs. mainland) in reproductive state. We used a similar model, but with two fixed factors, region (Baltic Sea islands vs. mainland) and reproductive state (sex expressing or not, excluding sporophytic samples), to test for effects on sex. To test whether differences between phenotypic and non-reproductive sex ratios differed between regions (i.e. whether sexes had different levels of expression in the two regions), we included the interaction between region and reproductive state in the latter model. The interaction was not significant, and we present the results of the model without the interaction. Finally, the selection of the study regions relied, in part, on sporophyte frequency estimates that were based on expert knowledge, indicative of sexual reproduction frequency. This motivated us also to quantify the differences in the level of successful sexual reproduction between regions in our sample sets. For both species, we tested whether the proportion of samples with sporophytes differed between regions (fixed factor) with generalized linear models with a binomial distribution and a log-link function. All statistical analyses were performed using Statistica v.13.5 (TIBCO_Software_Inc., 2018).

### Analyses of genetic diversity and composition

To answer the third and fourth questions, we identified haplotypes for each species based on the combined sequence information with the program TCS (Clement *et al.*, 2000). We estimated three measures of genetic diversity: effective number of haplotypes (Ne) and haplotype diversity (*H*), using GENALEX v.6.5 (Peakall and Smouse, 2006, 2012), and nucleotide diversity (π_n_), using ARLEQUIN v.3.5.2.2 (Excoffier and Lischer, 2010). We performed an AMOVA to partition genetic variation within and among samples of different sexes and regions and to calculate pairwise Φ_PT_, an analogue of *F*_ST_, to estimate genetic differentiation (GENALEX v.6.5; Peakall and Smouse, 2006, 2012). Finally, we contrasted nucleotide differentiation between samples of different sexes and regions, using corrected average pairwise differences allowing for equal nucleotide frequencies (Excoffier and Lischer, 2010). For both species, we first calculated potential differences in genetic diversity and genetic differentiation between the two geographical regions with different levels of realized sexual reproduction. When no differences were found, we pooled the samples across study regions and assessed differences and differentiation between males and females. Finally, when we revealed differences between regions and/or sexes, we analysed four categories of sex × region. Our null hypotheses were that there exist no differences between regions or sexes.

## RESULTS

### Sex ratios and reproductive state

In *D. trifarius*, 77 % of the 83 samples analysed from north Sweden were non-reproductive, 6 % were male, 16 % were female and 1 % bore sporophytes. The corresponding figures for south-central Sweden (165 samples), the region with regular sexual reproduction, were 55 % non-reproductive, 12 % male, 28 % female and 5 % sporophytic.

**Fig. 2.**
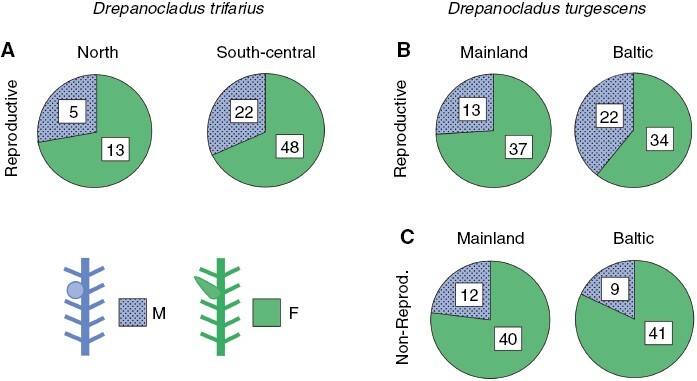
Proportions of male (M) and female (F) reproductive samples (phenotypic sex ratios) in *Drepanocladus trifarius* (A) and *D. turgescens* (B) and proportions of male and female non-reproductive samples in *D. turgescens* (C) in different regions in Scandinavia. Study regions for *D. trifarius*: North, northern Sweden; South-central, south-central Sweden; *D. turgescens*: Baltic, Baltic Sea islands; Mainland, mainland Scandinavia. Samples with both sexes were included as F and M: *D. trifarius* (south-central, four); *D. turgescens* (Baltic, three; mainland, two).

**Fig. 3.**
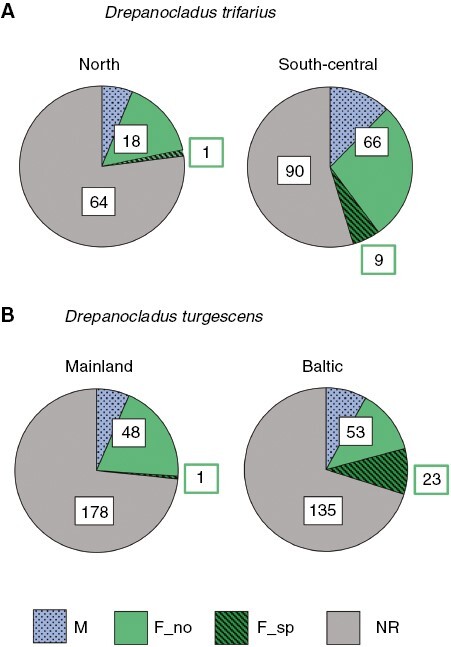
Reproductive state of samples of *Drepanocladus trifarius* (A; *n* = 248) and *D. turgescens* (B; *n* = 438) in different regions in Scandinavia. Study regions for *D. trifarius*: North, northern Sweden; South-central, south-central Sweden; *D. turgescens*: Baltic, Baltic Sea islands; Mainland, mainland Scandinavia. Abbreviations: F_no, samples with female sexual branches (perichaetia) but no sporophytes; F_sp, female samples with sporophytes; M, samples with male sexual branches (periogonia); NR, samples without reproductive structures. Sample numbers: [F_no + M, including samples with both male and female reproductive structures: *D. trifarius* (south-central, four); *D. turgescens* (Baltic, three; mainland, two), i.e. all reproductive nonsporophytic; samples], [sporophytic], [NR].

There was a female bias in the proportions of reproductive samples: relatively more samples were female than male in both north (13 vs. 5 samples) and south-central Sweden (44 vs. 18, plus 4 samples with both sexes), resulting in sex ratios <0.5 (Table 1A; Fig. 2A). Sex ratios did not differ between the two regions (Table 2). The proportion of samples that expressed sex were higher in south-central than in north Sweden (Table 2; Fig. 3A). Also, the proportion of samples with sporophytes was higher in south-central than north Sweden (Table 2; Fig. 3A).

**Table 1.** Deviations of regional sex ratios (proportion of male samples) from an expected balanced sex ratio of 0.5 in *Drepanocladus trifarius* (A) and *D. turgescens* (B, C) in regions with different levels of sexual reproduction in Scandinavia.

	Sex ratio	d.f.	χ^2^	*P*-value
Reproductive samples				
(A) *Drepanocladus trifarius*				
South-central Sweden (*n* = 70)	0.31	1	9.657	0.002
North Sweden (*n* = 18)	0.28	1	3.556	0.059
(B) *Drepanocladus turgescens*				
Mainland (*n* = 50)	0.26	1	11.520	<0.001
Baltic Sea islands (*n* = 56)	0.39	1	2.571	0.109
Non-reproductive samples				
(C) *Drepanocladus turgescens*				
Mainland (*n* = 52)	0.23	1	15.077	<0.001
Baltic Sea islands (*n* = 50)	0.18	1	20.480	<0.001

Abbreviations: d.f., degrees of freedom; *n*, number of samples analysed; *P*-value, significance level of χ^2^ tests for deviations from an expected balanced sex ratio (0.5). Samples with both sexes were counted as male and female for sex ratio assessments (*D. trifarius*, 4; *D. turgescens*, 5). Study regions (Fig. 1): *Drepanocladus trifarius*, south-central vs. north Sweden; *D. turgescens*, mainland Scandinavia vs. Baltic Sea islands Gotland and Öland. See Materials and Methods for details on datasets.

**Table 2.** Effects of region on sex ratio (proportions of male populations), reproductive state (reproductive vs. non-reproductive) and proportion of sporophytic samples in *Drepanocladus trifarius*; effects of region and reproductive state on sex ratio, and effect of region on reproductive state and proportion of sporophytic samples in *Drepanocladus turgescens*.

	*n*	d.f.	Log likelihood	χ^2^	*P*-value
Sex ratio					
*D. trifarius*	84				
Region		1	−52.645	0.204	0.652
*D. turgescens*	203				
Reproductive state		1	−117.035	5.066	0.024
Region		1	−116.757	0.555	0.456
Reproductive state					
*D. trifarius*	238				
Region		1	−149.433	10.176	0.001
*D. turgescens*	414				
Region		1	−229.722	0.596	0.440
Proportion sporophytic samples					
*D. trifarius*	248				
Region		1	−40.341	3.126	0.077
*D. turgescens*	438				
Region		1	−83.380	19.30	<0.001

Results of generalized linear models with binomial distribution and logit link function. Abbreviations: d.f., degrees of freedom; *n*, number of samples analysed; *P*-value significance level of log likelihood tests in the generalized linear model. Study regions (Fig. 1): *D. trifarius*, south-central vs. north Sweden; *D. turgescens*, mainland Scandinavia vs. Baltic See islands Gotland and Öland. ‘Reproductive state’ analyses excluded sporophytic samples (*D. trifarius*, 10; *D. turgescens*, 24). See Materials and Methods for details on datasets.

In *D. turgescens*, 73 % of the samples from mainland Scandinavia (184 samples) were non-reproductive, 7 % were male, 20 % were female and only one sample contained sporophytes (<1 %). On the Baltic Sea islands (254 samples), the region with more frequent sexual reproduction than the mainland, 70 % were non-reproductive, 8 % male, 13 % female and 9 % sporophytic.

Relatively more samples were female than male in both regions. This held true for both reproductive (35 vs. 11, plus two samples with both sexes, mainland; 31 vs. 19, plus three samples with both sexes, Baltic) and non-reproductive samples (40 vs. 12, mainland; 41 vs. 9, Baltic). The deviation from equality (ratio of 0.5) was not significant among the reproductive samples on the Baltic Sea islands (Table 1B, C; Fig. 2B). The two-factor model revealed a significant effect of reproductive state on sex ratio, whereas region was not significant (Table 2). The interaction effect between region and reproductive state on sex ratio was not significant (results not shown), although the difference in sex ratio between the reproductive and non-reproductive populations appeared greater on the Baltic Sea islands than on the mainland (Fig. 2B, C). The proportion of reproductive samples did not differ between the regions (Table 2). The proportion of *D. turgescens* samples that bore sporophytes was higher on the Baltic Sea islands than on the mainland (Table 2; Fig. 3B).

### Genetic diversity

In *D. trifarius*, genetic diversity did not differ between the north and south-central regions, whereas the female samples across study regions exhibited a higher diversity (Table 3A). The analyses of sex × region effects showed significant sex-related differences in genetic diversity in south-central Sweden, but small differences in north Sweden. Eleven of 17 (65 %) identified haplotypes were private to either region, and 9 of 17 (53 %) were private to either sex. Genetic variation occurred only within and not between the two study regions and different sexes. Pairwise comparisons of Φ_PT_ and the number of nucleotide differences between regions or the two sexes, respectively, were not significant (results not shown).

**Table 3.** Intraspecific genetic diversity in male and female samples of *Drepanocladus trifarius* (A) and *D. turgescens* (B) in two regions each with different levels of sexual reproduction in Scandinavia.

Region, sex and region × sex	*n*	Ne	*H*	π_n_ (s.d.)
(A) *Drepanocladus trifarius*				
South-central Sweden (SC)	44	4.4	0.775	0.0040 (0.0023)
North Sweden (N)	42	4.5	0.777	0.0032 (0.0016)
Females^A^	41	5.9	0.832	0.0044 (0.0023)
Males^A^	45	3.5	0.715	0.0030 (0.0016)
SC_Females^B^	20	6.7	0.850	0.0055 (0.0029)
SC_Males^B^	24	2.9	0.656	0.0028 (0.0015)
N_Females	21	4.3	0.766	0.0032 (0.0017)
N_Males	21	4.0	0.753	0.0033 (0.0018)
(B) *Drepanocladus turgescens*				
Mainland Scandinavia (MA)^C^	55	12.5	0.920	0.0017 (0.0009)
Baltic Sea islands (BA)^C^	33	9.3	0.893	0.0012 (0.0007)
Females	37	9.4	0.894	0.0020 (0.0011)
Males	31	12.5	0.920	0.0013 (0.0007)
BA_Females	15	5.8	0.827	0.0012 (0.0008)
BA_Males	16	6.1	0.836	0.0011 (0.0007)
MA_Females	22	7.8	0.872	0.0025 (0.0014)
MA_Males	15	9.0	0.889	0.0011 (0.0007)

Abbreviations: *H*, haplotype diversity; *n*, number of samples analysed; Ne, effective number of haplotypes; π_n_, nucleotide diversity (s.d.). Study regions (Fig. 1): *D. trifarius*, south-central vs. north Sweden; *D. turgescens*, mainland Scandinavia vs. Baltic Sea islands Gotland and Öland. Superscript letters A, B, C denote significant pairwise diversity differences based on Shannon’s information index: between samples A, *P* = 0.036; between samples B, *P* = 0.013; between samples C, *P* = 0.032 (Hutcheson’s *t*-test; https://www.dataanalytics.org.uk/comparing-diversity/).

In *D. turgescens*, genetic diversity in terms of Ne and *H* was higher on mainland Scandinavia than on the Baltic Sea islands. Male relative to female samples had a non-significantly higher genetic diversity, except for nucleotide diversity, which was higher in females than in males (Table 3B). The differential analyses of region × sex showed the same patterns (Table 3B). Eighty per cent (28 of 35) of the identified haplotypes were private to either region, and 79 % (23 of 29) were private to either sex. Although most of the genetic variation was found within the regions and sexes, the differentiation was significant both between regions (4 %) and between sexes (2 %; Table 4A). Pairwise comparisons of Φ_PT_ and the average number of pairwise nucleotide differences revealed differences between study regions and sexes (near-significant, *P* = 0.07, for sex differences in nucleotide diversity). The analyses of region × sex showed differences in Φ_PT_ between different sexes on the Baltic Sea islands but not on the mainland. Across regions, Φ_PT_ differed between the male population on the Baltic Sea islands and the populations of both sexes on the mainland. There was no differentiation in nucleotide differences between sexes within regions, whereas males and females differed in the average number of pairwise nucleotide differences between regions (Table 4B).

**Table 4.** (A) Genetic variation partitioning (AMOVA) within and between two regions with different levels of sexual reproduction in Scandinavia, and within and between sexes in *Drepanocladus turgescens*. (B) Pairwise Φ_PT_ values and average number of pairwise nucleotide differences between regions and sexes in *D. turgescens*.

(A)	d.f.	SS	MS	Estimated variance	Variation (%)
Between regions: BA, *n* = 33; MA, *n* = 55; Φ_PT_ = 0.035; *P* = 0.002					
Between regions	1	1.164	1.164	0.017	4
Within regions	86	40.018	0.465	0.465	96
Total	87	41.182	–	0.482	100
Between sexes: Females, *n* = 37; males, *n* = 31; Φ_PT_ = 0.020; *P* = 0.039					
Between sexes	1	0.790	0.790	0.010	2
Within sexes	66	30.799	0.467	0.467	98
Total	67	31.588	–	0.467	100
Between region × sex: BA_Females, *n* = 15; BA_Males, *n* = 16; MA_Females, *n* = 22; MA_Males, *n* = 15; Φ_PT_ = 0.054; *P* = 0.001					
Between regions	1	1.144	1.144	0.014	3
Between sexes	2	1.300	0.650	0.012	2
Within region × sex	64	29.145	0.455	0.455	95
Total	67	31.588		0.482	100

(B)	Φ_PT_	*P*-value	π_n_	*P*-value	
Between regions					
BA vs. MA	0.035	0.030	0.417	<0.001	
Between sexes					
Females vs. males	0.020	0.040	0.081	0.072	
Between region × sex					
BA_Females vs. BA_Males	0.056	0.040	0.104	0.200	
MA_Females vs. MA_Males	0.000	0.417	0.017	0.327	
BA_Males vs. MA_Males	0.059	0.008	0.525	<0.001	
BA_Males vs. MA_Females	0.092	<0.001	0.842	<0.001	
BA_Females vs. MA_Females	0.036	0.061	0.422	0.010	
BA_Females vs. MA_Males	0.020	0.220	0.184	0.060	

(A) Abbreviations: d.f., degrees of freedom; MS, mean squared deviations; *n*, number of samples analysed; SS, sums of squares. (B) Probability values (*P*) based on 9999 permutations for Φ_PT_ (indicative of genetic diferentiation between regions or sexes) and 1000 permutations for the average number of pairwise nucleotide differences. Study regions (Fig. 1): MA, mainland Scandinavia vs. BA, Baltic Sea islands Gotland and Oland.

## DISCUSSION

Samples of *D. trifarius* and *D. turgescens* were mostly female, and sex ratios did not differ between regions with different levels of realized sexual vs. clonal reproduction in either species. The proportion of samples that formed sexual structures was higher in the region with regular sexual reproduction in *D. trifarius*, whereas it did not differ between regions in *D. turgescens*. Sex ratios in *D. turgescens* were more strongly female biased in the non-reproductive samples, indicating that male plants expressed sex more often than females. Intraspecific genetic diversity did not differ between regions in *D. trifarius* but was regionally differentiated and higher in the region with rare sporophyte production in *D. turgescens*. There were sex-related differences in genetic diversity in both species. In conclusion, the regional frequency of sexual vs. clonal reproduction was not directly associated with regional sex ratios, proportions of sex-expressing samples and genetic diversity. Furthermore, the intersexual and interregional variation in reproductive traits and relationships with genetic diversity differed between the study species. Below, we interpret these findings in the context of the life histories of the species and historical factors.

### Sex ratio and reproductive state

Our results provide partial support for the first prediction, of a more strongly distorted phenotypic sex ratio in the region with rare than in the region with regular successful sexual reproduction for *D. turgescens*. The sex ratio of this species was female biased on the mainland and did not differ from equality on the Baltic Sea islands (Table 1). The second prediction, of a higher sex expression in the region with regular sexual reproduction, was supported for *D. trifarius*. For both species, the proportion of sporophytic samples was higher in the region with sexual populations than in the region with clonal populations. This suggests that fertilization is, to some degree, mate limited in both species and limited by male mates in *D. turgescens*. Mate-limited reproduction has previously been suggested based on negative correlations between strong female sex ratio bias and low sporophyte frequency (but see Boquete *et al.*, 2023) and confirmed experimentally in two mosses (Bisang *et al.*, 2004).

Our findings confirm the female gametophytic sex ratio bias in both *D. trifarius* and *D. turgescens* previously reported at the European and regional scales (Bisang *et al.*, 2006, 2014, 2015; Hedenäs *et al.*, 2010). It also aligns with the observed female dominance among adult populations in most dioicous bryophytes (Bisang and Hedenäs, 2005). However, it contrasts with the expectation of a balanced sex ratio following the segregation of sex chromosomes at meiosis (Bachtrog *et al.*, 2011; Supplementary data Table S1). Primary sex ratio distortions are widely reported in dioecious organisms (Haig, 2010). In the haploid-dioicous bryophytes, however, the situation for potential sex chromosome-linked drivers is symmetrical and would lead to an average primary sex ratio of 0.5, whereas cytoplasmic factors might be under selection to produce female-biased primary sex ratios (de Jong *et al.*, 2018). Plausible mechanisms for biased meiotic spore sex ratio variation in bryophytes seem restricted to differential survival of male and female spores (de Jong *et al.*, 2018; Carey *et al.*, 2021). The meiotic spore sex ratio has been assessed in few bryophyte species, and many results remain ambiguous because of large spore fractions that did not germinate and could not be sexed (reviewed by Bisang *et al.*, 2017). In *Drepanocladus lycopodioides*, related to our study species, the sex ratio among spores from three geographically separate populations, which germinated fully, was balanced (Bisang *et al.*, 2017). It is thus reasonable to assume that post-meiotic factors contribute significantly to the observed sex ratio bias across regions (Bisang and Hedenäs, 2005; Field *et al.*, 2013*a*).

Among the explanations referring to the post-meiotic stages that have been put forward to account for female dominance in bryophyte populations, it was postulated that males had lower levels of sex expression, i.e. fewer plants producing sexual organs than females (‘shy male hypothesis’; Mishler and Oliver, 1991). Previous studies have found indirect evidence both in favour and against this notion (Newton, 1971; Cronberg, 2002; Cronberg *et al.*, 2003; Stark *et al.*, 2010), but the direct relationship between the observed phenotypic and genotypic sex ratios was difficult to resolve. Methods to sex non-reproductive bryophytes were developed only in recent times (Korpelainen *et al.*, 2008, and references therein). Before this, bryophyte population sex ratios were inferred from counts of reproductive plants (reviewed by Bisang and Hedenäs, 2005; and e.g. Blackstock, 2015; Pereira *et al.*, 2016). In this study, we used a molecular marker-based approach to identify sex in non-reproductive populations of *D. turgescens* (Hedenäs *et al.*, 2016). We showed that male samples were relatively more often reproductive than female samples (effect of ‘reproductive state’; Table 2). This pattern is in agreement with the overall notion that differential sex expression can contribute to a deviating phenotypic relative to genetic sex ratio. Our data point in the opposite direction to that postulated in the ‘shy male hypothesis’. Recently, sex ratios in adult non-reproductive populations were reported for a few species, commonly exhibiting a female bias. Some studies indicated overall sex-independent expression rates (Hedenäs *et al.*, 2010; Bisang and Hedenäs, 2013; Bisang *et al.*, 2015, 2020), but in others, expression levels varied between sexes and regions (present study; Baughman *et al.*, 2017; Ekwealor *et al.*, 2022).

In the present study, the larger fraction of reproductive than non-reproductive male samples in *D. turgescens*, reflecting a higher degree of sex expression in males than in females, appears to be manifested mainly on the Baltic Sea islands but much less so on the mainland (Fig. 2B vs. C). Despite this noticeable difference, the interaction effect between region and reproductive state was not significant, possibly because males were rare overall. However, relatively more male reproductive samples on the Baltic Sea islands than on the mainland were also reflected in the balanced phenotypic sex ratio for the former compared with the female-biased phenotypic sex ratio for the latter (Table 1B; Fig. 3B). The Baltic Sea surrounding the islands contributes to longer growth seasons and relatively higher autumn temperatures compared with the mainland (Sjörs, 1999), creating an environment that could be favourable to the formation of sexual organs in male *D. turgescens*. Environmental effects on formation of gametangia (sex expression) are well known in bryophytes (e.g. Chopra and Bathla, 1983; Rydgren and Økland, 2002), and geographical variation in sex phenotypes and sex expression have been demonstrated repeatedly in different plant groups (Benassi *et al.*, 2011; Yakimowski and Barrett, 2014; Blackstock, 2015; Pereira *et al.*, 2016). Sex expression rates differed between different microenvironments in the desert moss *Syntrichia caninervis* (Baughman *et al.*, 2017; Ekwealor *et al.*, 2022) and varied across Europe along macroscale environmental gradients in the perennial pleurocarpous moss *Pseudoscleropodium purum* (Boquete *et al.*, 2023). However, little is known about environmental effects on genotypic sexes in bryophytes (Bisang *et al.*, 2015, 2020).

The two study species have in common that they reproduce clonally during long periods and in extensive areas and that sexual reproduction is spatially restricted and episodic. Nevertheless, the species differ in ecology, distribution and reproductive performance. The common life-history traits, differences in the life-history traits, and historical factors are important to interpret our finding that the regional frequency of successful sexual reproduction was not directly associated with variation in other reproductive traits and genetic diversity. Bryophyte spores are easily wind dispersed and provide distance-independent colonization probabilities (Lönnell *et al.*, 2012; Patiño and Vanderpoorten, 2018). Successful establishment from bryophyte spores, however, might represent a bottleneck for many species even in the case of sufficient spore rain, for example owing to substrate limitation, specific germination requirements or local competition (Lönnell *et al.*, 2014). Consequently, many pleurocarpous mosses, such as *D. trifarius* and *D. turgescens*, have generation lengths of several decades (Bergamini *et al.*, 2019) and might sustain clonal populations over thousands of years (Hedenäs, 2014; Vitt and House, 2021). Clonal growth affects sex ratios by altering the number and spatial distribution of reproductive units, accentuating the difference in sex expression levels between sexes and regions and, potentially, increasing the distance between mates. Furthermore, if fertilization fails, female reproductive investment is confined to the prezygotic stage. This is considerably lower or zero in non-expressing plants than postzygotic allocation to a sporophyte that depends nutritionally on the gametophyte (Bisang and Ehrlén, 2002; Rydgren and Økland, 2003; Bisang *et al.*, 2006; Haig and Wilczek, 2006). If females instead allocate more resources to vegetative growth, intersexual distances and constraints for mates to meet will increase. In situations with restricted sexual recruitment, a sex ratio bias can remain over long periods (Barrett, 2015; Hedenäs *et al.*, 2021). For several bryophyte species, the time available for colonization after the Last Glacial Maximum was suggested to contribute to skewed population sex ratios (Alonso-García *et al.*, 2020; Bisang *et al.*, 2020; Boquete *et al.*, 2023). In vascular herbaceous plants, clonality was associated with female-biased sex ratios (Charpentier, 2002; Barrett, 2015). Clonal species exhibited greater heterogeneity in sex ratios than non-clonal species as a result of their slower approach to equilibrium and of strong founder effects (Field *et al.*, 2013*b*). The strong functional link between life-history in terms of life span and mating systems was emphasized recently in flowering plants (Pannell, 2021). For our study species, we infer that a long life span of the populations, strong ability to spread clonally and limited recruitment possibilities contributed to the strong female sex ratio bias also in regions where spores are produced. This interpretation concurs with the balanced ratio among reproductive male and female samples of *D. turgescens* on the Baltic Sea islands owing to a higher degree of sex expression in males. Lastly, reproductive traits that have evolved in ancestors might show limited regional variation (Crawford *et al.*, 2009; Ma *et al.*, 2020). For the study species, we have shown previously that family position as a proxy for phylogenetic relatedness explained 56 or 13 %, respectively, of the variation in phenotypic sex ratios and sex expression (Bisang *et al.*, 2014).

### Genetic diversity

Genetic diversity in the two species was comparable to that in previously studied bryophytes (e.g. Szövényi *et al.*, 2006; Hedenäs, 2017). Overall, genetic diversity (*H*, π_n_), the number of effective haplotypes and number of private haplotypes per region were higher in *D. turgescens* than in *D. trifarius*. This could have resulted from glacial survival in small, isolated refugia and related genetic drift in *D. turgescens* (Frankham *et al.*, 2002). This species frequently occurs in Arctic climatic conditions, unlike *D. trifarius* (Hedenäs, 2014). Furthermore, many rich fens in south Sweden, which are common habitats of the latter species, are suffering from environmental deterioration (Gunnarsson and Löfroth, 2009), which might have impacted genetic diversity in *D. trifarius* more recently.

Intraspecific genetic diversity did not differ between regions in *D. trifarius*. However, in *D. turgescens* it was lower on the Baltic Sea islands, the region with episodically ample sporophyte production, than on mainland Scandinavia (Table 3). Our data thus do not support the third prediction of lower genetic diversity in areas with rare rather than regular sexual reproduction. In *D. turgescens*, the proportion of genetic variation between regions was significant, albeit limited (4 %; Table 4), and was in the lower range of interregional differentiation observed in other plant species (Zhang *et al.*, 2005; Hedenäs, 2017). Previous studies of different plant species reported that genetic variation could also be retained in predominantly clonal populations, depending on the colonization history of a species (e.g. Wang *et al.*, 2004; Karlin *et al.*, 2012; Scalone and Albach, 2012; Meloni *et al.*, 2013; Chrzanowska *et al.*, 2016). ‘Conserved diversity’ might account, in part, for the low between-region differentiation in our target species. Moreover, *D. turgescens* exhibits overall a higher sporophyte frequency than *D. trifarius* and intermittently produces many easily wind-dispersed spores (Miller, 1980; Bisang *et al.*, 2014; Hedenäs and Bisang, 2019*a*). *Drepanocladus trifarius* mainly occurs in stable habitats, such as deep fens, whereas *D. turgescens* occupies dynamic environments, such as temporarily wet, shallow depressions, which provide gaps for colonization recurrently (Hedenäs, 2003). The extent of local *D. turgescens* populations has been observed to fluctuate strongly among years, probably in response to variable weather conditions (pers. obs. IB and LH; Hedenäs and Bisang, 2019*a*). This species spreads vegetatively by means of easily detached shoot apices, which facilitates dispersal and re-establishment after years with unfavourable weather conditions or local disturbances (Longton and Schuster, 1983; Hedenäs, 2003). Disturbance events, based on specific dispersal modes and abilities of this species, can allow for genetic mixing within and across regions (e.g. Eckert *et al.*, 2016). Genetic data suggested that the mainland and the Baltic Sea island populations have different postglacial origins, accounting, in part, for the different diversities in the two regions, and that some dispersal northwards has occurred between 250 and 500 years BP (Hedenäs, 2014).

We found sex-related differences in genetic variation in both species. In *D. trifarius*, genetic diversity (Ne, *H* and π_n_) was higher in female than in male samples in south-central Sweden, supporting our fourth prediction, whereas the difference between male and female samples of *D. turgescens* was not significant (Table 3). In a previous study, females of *D. trifarius* across its European distribution range also displayed higher diversity than males (Hedenäs and Bisang, 2013). In *D. turgescens*, there was a small but significant between-sex part of the genetic variation, which appeared to be driven by the distinctness of males on the islands (Table 4). In the hornwort *Nothoceros aenigmaticus*, spatially isolated male and female populations were strongly differentiated genetically (Alonso-García *et al.*, 2020). In both *D. trifarius* and *D. turgescens*, males were much rarer than females, but genetic diversity was not lower than in females in the latter. As mentioned above, *D. turgescens* exhibits overall a higher sporophyte frequency than *D. trifarius*. We propose that the recurrent, albeit sporadic, spore production and establishment possibilities in *D. turgescens* relative to the predominating clonal reproduction in *D. trifarius* in more stable habitats contribute to genetic variation also in the rarer sex (Eckert, 2016).

Both sex-related genetic differences and genetic structuring or lack thereof have been reported in seed plants (e.g. Dering *et al.*, 2016; Zhai *et al.*, 2016, and references therein). Loss of genetic variation can happen when populations pass bottlenecks, for example during glacial or other types of harsh periods, which is more likely for the sex with fewer occurrences (Frankham *et al.*, 2002; Hedenäs and Bisang, 2013; Kyrkjeeide *et al.*, 2014; Alonso-García *et al.*, 2020). Along this line of reasoning, Baughman *et al.* (2017) explained low genetic diversity in males of the desert moss *Syntrichia caninervis*. He hypothesized that males should have a higher mortality in severe conditions because they allocate more resources prezygotically in sexual reproduction than females, which could result in genetic deprivation. However, newer evidence showed that non-reproductive males occurred in similar frequencies to females in exposed microsites (Ekwealor *et al.*, 2022), and male rarity was probably attributable to lower male sex expression. Finally, restricted sexual recruitment in unfavourable environmental conditions might facilitate the evolution of ‘genetic sterility’ through selection against traits involved in sex. This can occur, for example, when sexual reproduction incurs a higher fitness cost than clonal propagation (trade-off) and could be enhanced by additional advantages of clonality in the given surroundings (Eckert, 2002, 2016; Carey *et al.*, 2021).

### Conclusions

Our results provided only partial support for the predictions that variation in sex ratios, reproductive state and intraspecific genetic differentiation are associated with successful sexual reproduction at the regional level. We showed that reproductive trait and intraspecific variation patterns differed between sexes. In addition to the degree of sexual vs. clonal reproduction, we discuss how the following factors can contribute to shaping of the regional variation in reproductive traits and intraspecific genetic diversity: species-specific life-history traits, such as population longevity or recruitment frequency; sex-specific performance; environmental conditions, such as disturbance frequency or seasonal temperature regimes; the response of demographic processes to the environment at the level of the individual; and historical factors. In persistent populations with extensive clonality, the imbalance in reproductive traits is likely to increase over time, further constraining the chances of fertilization. This entails that the effect of stochastic events and historical factors, such as migration history or ancestry, are maintained over long time periods and might be more important than effects of the frequency of sexual reproduction. Moreover, bryophyte lineages and groups within lineages can be characterized by principal growth forms (La Farge-England, 1996), which correspond, in part, to their phylogeny. We argue that generalizations across these major growth forms are too simplified to assess bryophyte reproductive patterns.

To increase the understanding of bryophyte sex ratios, other reproductive traits and their interspecific and spatial variation, we call for more detailed population-level studies, for a more intense sampling of non-reproductive plants to assess primary sex ratios and performance during prezygotic life cycle phases and for differentiating sex responses from sex expression responses. The present rapid development of genomics facilitates the development of sex identification methods for non-reproductive samples. Genomic data will also allow for a higher resolution of intraspecific genetic variation that can be compared with reproductive patterns (e.g. Carey *et al.*, 2021). Such knowledge will improve our understanding of evolutionary processes. Finally, historical factors should be considered to elucidate reproductive patterns, especially in long-lived species. Such extended data on the reproductive biology of haploid-dominated plants will provide crucial information to help us understand the complexity of plant reproductive patterns.

## SUPPLEMENTARY DATA

Supplementary data are available online at https://academic.oup.com/aob and consist of the following. Table S1: bryophyte life cycle and bryophyte-specific terminology. Table S2: studied material of *Drepanocladus trifarius* and *D. turgescens*. Table S3: sample localities, associated data and GenBank accession numbers for *Drepanocladus trifarius* and *D. turgescens* studied for genetic variation; and samples used for molecular sex identification in *D. turgescens*. Figure S1: sample locations of *Drepanocladus trifarius*. Figure S2: sample locations of *Drepanocladus turgescens*.

mcad045_suppl_Supplementary_Figure_S1-S2

mcad045_suppl_Supplementary_Table_S1

mcad045_suppl_Supplementary_Table_S2

mcad045_suppl_Supplementary_Table_S3

mcad045_suppl_Supplementary_Data
