# Supplementary material for: Life-history characteristics and historical factors are important to explain regional variation in reproductive traits and genetic diversity in perennial mosses
Source: Ann Bot. 2023 Mar 16;132(1):29–42. doi: 10.1093/aob/mcad045 (PMC10550275; doi:10.1093/aob/mcad045)
Supplement: mcad045_suppl_Supplementary_Table_S1 [file mcad045_suppl_supplementary_table_s1.docx]

Supplementary Material, Table S1: Bryophyte life cycle and bryophyte specific terminology

Bisang, Ehrlén & Hedenäs:

**Life history characteristics and historical factors are important to explain regional variation in reproductive traits and genetic diversity in perennial mosses**

Mosses are one of three lineages that constitute the monophyletic bryophytes (Su *et al.*, 2021), which share a haploid-dominant life cycle. Sex organs are formed on the gametophyte. In mosses, the gametangia, carrying egg cells or spermatozoids, and the surrounding specialized photosynthetic leaves~~,~~ form reduced sexual branches, called **perichaetia** in females and **perigonia** in males. Spermatozoids with limited motility require a water film to reach and fertilize the sessile ovum (Haig, 2016). The **sporophyte** develops from the zygote, remains attached to the maternal gametophyte during its lifetime, and the **spores** shed from the sporophyte germinate to produce new **gametophytes**. Sex determination occurs at meiosis in the sporophyte rather than at syngamy, as in flowering plants and many animal groups (Haig, 2016). In dioicous species, the sex-determining loci on the **heteromorphic U and V chromosomes** separate at meiosis and spores and gametophytes are either male or female (Bachtrog *et al.*, 2011; Renner *et al.*, 2017). Even gametophytes that do not form gametangia carry a genotypic sex based on sex determination at meiosis. We term individuals, patches and populations without sexual organs, i.e., without sex expression, ‘**non-reproductive**’ rather than ‘sterile’ (Bisang *et al.*, 2020). Most bryophytes lack evident secondary morpho-anatomical sex characteristics, which prevents sex identification without molecular methods in non-reproductive plants (e.g., Korpelainen *et al.*, 2008). Here, we distinguish between **phenotypic sex ratios** (based on reproductive populations) and **sex ratios of non-reproductive plants** identified with molecular methods. “True” or genotypic sex ratios are a combination of these two types, and are independent of reproductive state (Bisang *et al.*, 2020: Fig. 1).

LITERATURE CITED

**Bachtrog D, Kirkpatrick M, Mank JE, McDaniel SF, Pires JC, Rice W et al. 2011.** Are all sex chromosomes created equal? *Trends in Genetics* 27: 350-357. doi:10.1007/s00442-017-3966-5.

**Bisang I, Ehrlén J, Hedenäs L.** **2020**. Sex expression and genotypic sex ratio vary with region and environment in the wetland moss *Drepanocladus lycopodioides*. *Botanical Journal of the Linnean Society* **192**: 421-434. doi:10.1093/botlinnean/boz063.

**Haig D.** **2016**. Living together and living apart: the sexual lives of bryophytes. *Philosophical Transactions of the Royal Society B: Biological Sciences* **371**. doi:10.1098/rstb.2015.0535.

**Korpelainen H, Bisang I, Hedenäs L, Kolehmainen J.** **2008**. The first sex-specific molecular marker discovered in the moss *Pseudocalliergon trifarium*. *Journal of Heredity* **99**: 581–587. doi:10.1093/jhered/esn036.

**Renner SS, Heinrichs J, Sousa A.** **2017**. The sex chromosomes of bryophytes: Recent insights, open questions, and reinvestigations of *Frullania dilatata* and *Plagiochila asplenioides*. *Journal of Systematics and Evolution* **55**: 333-339. doi:10.1111/jse.12266.
