# Supplementary material for: Life-history characteristics and historical factors are important to explain regional variation in reproductive traits and genetic diversity in perennial mosses
Source: Ann Bot. 2023 Mar 16;132(1):29–42. doi: 10.1093/aob/mcad045 (PMC10550275; doi:10.1093/aob/mcad045)
Supplement: mcad045_suppl_Supplementary_Table_S3 [file mcad045_suppl_supplementary_table_s3.docx]

Supplementary Material, Table S3

Bisang, Ehrlén & Hedenäs:

**Life history characteristics and historical factors are important to explain regional variation in reproductive traits and genetic diversity in perennial mosses**

**(A):** Sample localities, associated data and GenBank accession numbers for *Drepanocladus trifarius* and *D. turgescens* studied for genetic variation. Data format: **Sample No.:** Locality; Coll. Year, *Collector* [*collector’s No.*] (*LH* = L. Hedenäs); Herbarium: herbarium registration No.; GenBank Accession numbers for ITS, *gpd*, *rpl*16.

**(B):** Samples used for molecular sex identification in *D. turgescens*. Samples studied for both sex identification and genetic variation have the sample numbers for the latter (sometimes another PT number) in parentheses. Data format: **Sample No._SEX (F = female; M = male):** Locality; Coll. Year, *Collector* [*collector’s No.*] (*LH* = L. Hedenäs); Herbarium: herbarium registration No.

**A**

***Drepanocladus trifarius* (F. Weber & D. Mohr) Broth.: PT9A:** Sweden. Närke, Kil, lake Igeltjärn; 2005, *LH & A.Kooijman*; S: B104844; MK456067; MK467118; MK466432. **PT16:** Sweden. Pite Lappmark, Bockmyran, NNE Hedberg; 2000, *C.Jonsson*; S: B110749 ; KC601905; KC601867; KC601951. **PT17:** Sweden. Jämtland, Åre, 2 km E Storlien railway stn.; 2006, *I.Bisang, LH*; S: B112892; KC601906; KC601868; KC601952. **PT19:** Sweden. Jämtland, Åre, 2 km E Storlien railway stn.; 2006, *I.Bisang,* *LH*; S: B112894; KC601907; KC601869; KC601953. **PT20:** Sweden. Jämtland, Åre, 1.5 km W Lake Visjön; 2006, *I.Bisang,* *LH*; S: B112895; KC601908; KC601870; KC601954. **PT23:** Sweden. Härjedalen, Linsell, Glöte, Dyckessjön; 2007, *LH et al.*; S: B121274; KC601909; KC601871; KC601955. **PT24:** Sweden. Jämtland, Åre, Tännforsmyren; 2007, *LH et al.*; S: B121967; KC601910; KC601872; KC601956. **PT25:** Sweden. Jämtland, Åre, Enafors, Oppdalsvallen; 2007, *LH et al.*; S: B121968; KC601911; KC601859; KC601957. **PT26:** Sweden. Jämtland, Åre, Visjövalen; 2007, *LH et al.*; S: B121969; KC601912; KC601873; KC601958. **PT27:** Sweden. Jämtland, Åre, N Saxtjärnen; 2007, *LH et al.*; S: B121970; KC601913; KC601874; KC601959. **PT29:** Sweden. Jämtland, Åre, Storvallen, N Tävlan; 2007, *LH et al.*; S: B121911; KC601914; KC601875; KC601960. **PT31:** Sweden. Jämtland, Åre, NW Saxsjön; 2007, *LH et al.*; S: B121913; KC601915; KC601876; KC601961. **PT35:** Sweden. Uppland, Fasterna, Kornamosse; 1997, *K.Hylander 1993*; S: B124821; KC601917; KC601877; KC601963. **PT36:** Sweden. Härjedalen, Sveg, S Rossstjärnarna; 1989, *LH HD89-106*; S: B93295; KC601918; KC601878; KC601964. **PT37:** Sweden. Uppland, Norrtälje, Edebo; 1996, *K.Hylander 1949*; S: B94538; MK456068; MK467119; MK466433. **PT38:** Sweden. Uppland, Norrtälje, Singö, Stornotsand; 1996, *K.Hylander 1937*; S: B94539; MK456069; MK467120; MK466434. **PT39:** Sweden. Härjedalen, Tännäs, S Ramundberget; 2007, *LH*; S: B121277; KC601919; KC601861; KC601965. **PT40:** Sweden. Småland, Källeryd, Långelaggen; 1996, *LH & A.Kooijman*; S: B1116; KC601920; KC601862; KC601966. **PT42:** Sweden. Härjedalen, Ljusnedal, W Holmvallen; 2007, *LH*; S: B121279; KC601921; KC601879; KC601967. **PT43:** Sweden. Härjedalen, Tännäs, N Bruksvallarna; 2007, *LH*; S: B121278; KC601922; KC601863; KC601968. **PT44:** Sweden. Härjedalen, Ljusnedal, N Axhögvallen; 2007, *LH*; S: B121275; KC601923; KC601880; KC601969. **PT46:** Sweden. Pite Lappmark, Arjeplog, Mt. Ailesvare; 2006, *LH et al.*; S: B114435; KC601924; KC601864; KC601970. **PT47:** Sweden. Pite Lappmark, Arjeplog, Mt. Skärrim; 2006, *LH et al.*; S: B114018; KC601925; KC601865; KC601971. **PT48:** Sweden. Pite Lappmark, Arjeplog, Mt. Skärrim; 2006, *LH et al.*; S: B114017; KC601926; KC601881; KC601972. **PT49:** Sweden. Lule Lappmark, Lule-Akojaure, NO Nausta; 1996, *M.Norin*; S: B110751; MK456070; MK467121; MK466435. **PT50:** Sweden. Lule Lappmark, Saltoape, NW Varjisträsk; 1996, *M.Norin*; S: B110750; MK456071; MK467122; MK466436. **PT51:** Sweden. Lule Lappmark, Kairaträsket, E Tårrajaure; 1996, *S.Westerberg*; S: B110753; MK456072; MK467123; MK466437. **PT52:** Sweden. Torne Lappmark, Jukkasjärvi, S Kokkajärvi; 2002, *LH*; S: B73400; KC601927; KC601882; KC601973. **PT53:** Sweden. Torne Lappmark, Jukkasjärvi, W Paddustieva; 2002, *LH*; S: B73402; KC601928; KC601883; KC601974. **PT54:** Sweden. Torne Lappmark, Jukkasjärvi, SSW Tuore Sitsavaara; 2002, *LH*; S: B73436; KC601929; KC601884; KC601975. **PT55:** Sweden. Torne Lappmark, Jukkasjärvi, SSW Paddustieva; 1997, *LH*; S: B42919; KC601930; KC601885; KC601976. **PT59:** Öland, Böda, Svartvikskärret; 1993, *K.Hylander 252*; S: B64105; KC601931; KC601866; KC601977. **PT60:** Sweden. Jämtland, Frostviken, E Lillälven; 1986, *LH*; S: B124833; KC601932; KC601886; KC601978. **PT61:** Sweden. Jämtland, Frostviken, Hovdbäcken; 1988, *LH J88-76*; S: B124834; KC601933; KC601887; KC601979. **PT64:** Sweden. Jämtland, Alanäs, Lillviken-Lidsjöberg; 1997, *LH & A.Kooijman*; S: B1417; KC601934; KC601888; KC601980. **PT66:** Sweden. Jämtland, Frostviken, Autjovallennjuona; 1997, *LH & A.Kooijman*; S: B1133; KC601935; KC601889; KC601981. **PT70:** Sweden. Jämtland, Åre, Mt. Snasahögarna; 2002, *LH*; S: B73973; KC601936; KC601890; KC601982. **PT76:** Sweden. Jämtland, Åre, Mt. Skurdalshöjden; 2006, *I.Bisang, LH*; S: B112902; KC601937; KC601891; KC601983. **PT77:** Sweden. Jämtland, Åre, Mt. Skurdalshöjden; 2006, *I.Bisang, LH*; S: B112903; KC601938; KC601892; KC601984. **PT81:** Sweden. Dalsland, Färgelanda, Tollsvattnet-Hjärtsäter; 1952, *H.Sjörs*; S: B97418; MK456073; MK467124; MK466438. **PT82:** Sweden. Västmanland, Viker, Hovmanstorpasjön; 1961, *N.Hakelier*; S: B97491; MK456074; MK467125; MK466439. **PT83:** Sweden. Västmanland, Viker, Bromossen; 1961, *N.Hakelier*; S: B97493; MK456075; MK467126; MK466440. **PT84:** Sweden. Västmanland, Linde, Mårdshyttan; 1961, *N.Hakelier*; S: B97494; MK456076; MK467127; MK466441. **PT85:** Sweden. Västmanland, Viker, Ormtjärn; 1954, *N.Hakelier*; S: B97498; MK456077; MK467128; MK466442. **PT86:** Sweden. Västmanland, Viker, W Älvlången (Röjängen); 1958, *N.Hakelier*; S: B97499; MK456078; MK467129; MK466443. **PT87:** Sweden. Uppland, Söderby-Karl, SW Älvsjö; 1996, *LH & K.Hylander*; S: B1117; MK456079; MK467130; MK466444. **PT88:** Sweden. Dalsland, Järbo, Mölnetjärnet; 2008, *LH & C.Persson*; S: B133439; MK456080; MK467131; MK466445. **PT89:** Sweden. Dalsland, Steneby, E Björkhagen; 2008, *LH & C.Persson*; S: B133441; MK456081; MK467132; MK466446. **PT90:** Sweden. Dalsland, Steneby, Kölvattnet; 2008, *LH & C.Persson*; S: B133442; MK456082; MK467133; MK466447. **PT250:** Sweden. Södermanland, Åker, Strålsjömossen; 1962, *A.Johansson*; S: B94540; KC601943; KC601897; KC601989. **PT251:** Sweden. Närke, Vintrosa, N Lannafors; 1961, *N.Hakelier*; S: B97426; KC601944; KC601898; KC601990. **PT252:** Sweden. Jämtland, Åre, E Handöl; 2005, *LH et al.*; S: B104736; KC601945; KC601899; KC601991. **PT253:** Sweden. Jämtland, Ragunda, NW Ammer; 2012, *LH*; S: B193537; KC601946; KC601900; KC601992. **PT254:** Sweden. Lycksele Lappmark, Stensele, SW Slussfors; 2012, *LH*; S: B193552; KC601947; KC601901; KC601993. **PT1400:** Sweden. Gotland, Hejnum, Hejnum hällar; 2015, *LH*; S: B220541; MK456083; MK467134; MK466448. **PT1401:** Sweden. Småland, Kärda, Björnakullakärret; 1975, *T.Hallingbäck TH 612*; S: B217261; MK456084; MK467135; MK466449. **PT1402:** Sweden. Lycksele Lappmark, Lycksele, W Storbacksjön; 1987, *LH*; S: B93106; MK456085; MK467136; MK466450. **PT1403:** Sweden. Lycksele Lappmark, Tärna, Atofjället; 2012, *LH*; S: B193548; MK456086; MK467137; MK466451. **PT1404:** Sweden. Lycksele Lappmark, Stensele, SW Slussfors; 2012, *LH*; S: B193550; MK456087; MK467138; MK466452. **PT1405:** Sweden. Lycksele Lappmark, Tärna, Raavriedenjuenie; 2016, *LH*; S: B237325; MK456088; MK467139; MK466453. **PT1406:** Sweden. Pite Lappmark, Arjeplog, Vävkkáråhto; 2015, *LH et al.*; S: B223727; MK456089; MK467140; MK466454. **PT1407:** Sweden. Pite Lappmark, Arjeplog, E Mavas; 2015, *LH et al.*; S: B224565; MK456090; MK467141; MK466455. **PT1408:** Sweden. Pite Lappmark, Arjeplog, Mt. Ákháris; 2015, *LH et al.*; S: B224592; MK456091; MK467142; MK466456. **PT1409:** Sweden. Pite Lappmark, Arjeplog, Mt. Guotko; 2015, *LH et al.*; S: B224607; MK456092; MK467143; MK466457. **PT1410:** Sweden. Torne Lappmark, Jukkasjärvi, E Perälompolo; 2002, *LH*; S: B73401; MK456093; MK467144; MK466458. **PT1411:** Sweden. Åsele Lappmark, Dorotea, SE Harrsjö; 2004, *LH*; S: B93190; MK456094; MK467145; MK466459. **PT1412A:** Sweden. Lule Lappmark, Gällivare, Muddusape; 1991, *LH*; S: B92649; MK456095; MK467146; MK466460. **PT1413A:** Sweden. Lule Lappmark, Gällivare, Torisjoki; 2017, *LH*; S: B253208; MK456096; MK467147; MK466461. **PT1414A:** Sweden. Lule Lappmark, Gällivare, Torisjoki; 2017, *LH*; S: B253217; MK456097; MK467148; MK466462. **PT1415A:** Sweden. Lule Lappmark, Jokkmokk, ENE Haraudden; 2017, *LH*; S: B253143; MK456098; MK467149; MK466463. **PT1416A:** Sweden. Pite Lappmark, Arjeplog, Mt. Skärrim; 2017, *LH et al.*; S: B258098; MK456099; MK467150; MK466464. **PT1417A:** Sweden. Pite Lappmark, Arjeplog, Mt. Själbmá; 2017, *LH et al.*; S: B258185; MK456100; MK467151; MK466465. **PT1418A:** Sweden. Pite Lappmark, Arjeplog, Mt. Själbmá; 2017, *LH et al.*; S: B258208; MK456101; MK467152; MK466466. **PT1419A:** Sweden. Pite Lappmark, Arjeplog, Mt. Stuor-Jiervas; 2017, *LH et al.*; S: B258271; MK456102; MK467153; MK466467. **PT1420A:** Sweden. Pite Lappmark, Arjeplog, Mt. Tjidtják; 2017, *LH et al.*; S: B258382; MK456103; MK467154; MK466468. **PT1421A:** Sweden. Pite Lappmark, Arjeplog, Mt. Tjidtják; 2017, *LH et al.*; S: B258522; MK456104; MK467155; MK466469. **PT1422A:** Sweden. Pite Lappmark, Arjeplog, Mt. Skärrim; 2017, *LH et al.*; S: B258092; MK456105; MK467156; MK466470. **PT1423A:** Sweden. Pite Lappmark, Arjeplog, Mt. Tjápkávárddo; 2017, *LH et al.*; S: B258433; MK456106; MK467157; MK466471. **PT1424A:** Sweden. Pite Lappmark, Arjeplog, Mt. Tjápkávárddo; 2017, *LH et al.*; S: B258428; MK456107; MK467158; MK466472. **PT1425A:** Sweden. Pite Lappmark, Arjeplog, NW Tjápkávárddo; 2017, *LH et al.*; S: B258547; MK456108; MK467159; MK466473. **PT1426A:** Sweden. Torne Lappmark, Jukkasjärvi, Vássečohka; 2017, *LH*; S: B256512; MK456109; MK467160; MK466474. **PT1427A:** Sweden. Torne Lappmark, Jukkasjärvi, Rákkasorda; 2017, *LH*; S: B256529; MK456110; MK467161; MK466475. **PT1428A:** Sweden. Torne Lappmark, Jukkasjärvi, S Låktatjåkka; 2017, *LH*; S: B256696; MK456111; MK467162; MK466476. **PT1429A:** Sweden. Lycksele Lappmark, Lycksele, Pausele; 2018, *LH*; S: B279851; MK456112; MK467163; MK466477. **PT1430A:** Sweden. Lycksele Lappmark, Stensele, ENE Forsmark; 2018, *LH*; S: B279880; MK456113; MK467164; MK466478. **PT1431A:** Sweden. Lycksele Lappmark, Stensele, Stormyrtorp; 2018, *LH*; S: B279901; MK456114; MK467165; MK466479. ***Drepanocladus turgescens* (T. Jensen) Broth.: P07:** Sweden. Gotland, Stenkyrka; 2001, *LH*; S: B62357; FJ535774, KJ657787, FJ535792. **PT1002:** Sweden. Öland, Eriksöre; 2010, *LH*; S: B174624; KJ657878, KJ657774, KJ658022. **PT1003:** Sweden. Gotland, Bunge; 1996, *LH*; S: B1126; KJ657891, KJ657788, KJ658035. **PT1004:** Sweden. Öland, Resmo; 2010, *LH*; S: B174629; KJ657879, KJ657775, KJ658023. **PT1005:** Sweden. Öland, Stenåsa; 2010, *LH*; S: B174790; KJ657880, KJ657776, KJ658024. **PT1006:** Sweden. Torne Lappmark, Torneträsk area; 1997, *LH*; S: B39204; KJ657940, KJ657836, KJ658084. **PT1007b:** Sweden. Gotland, Othem; 2011, *LH*; S: B184192; KJ657892, KJ657789, KJ658036. **PT1008:** Sweden. Öland, Räpplinge; 2011, *LH & I.Bisang*; S: B184235; KJ657881, KJ657777, KJ658025. **PT1010:** Sweden. Öland, Räpplinge; 2011, *LH & I.Bisang*; S: B184239; KJ657882, KJ657778, KJ658026. **PT1014:** Sweden. Öland, Torslunda; 2011, *LH & I.Bisang*; S: B184243; KJ657883, KJ657779, KJ658027. **PT1015:** Sweden. Öland, Resmo; 2011, *LH & I.Bisang*; S: B184241; KJ657884, KJ657780, KJ658028. **PT1019:** Sweden. Öland, Stenåsa; 2011, *LH & I.Bisang*; S: B184246; KJ657885, KJ657781, KJ658029. **PT1022:** Sweden. Öland, Vickleby; 2011, *LH & I.Bisang*; S: B184240; KJ657886, KJ657782, KJ658030. **PT1024:** Sweden. Gotland, Fleringe; 2011, *LH*; S: B184254; KJ657893, KJ657790, KJ658037. **PT1025:** Sweden. Gotland, Fleringe; 2011, *LH*; S: B184253; KJ657894, KJ657791, KJ658038. **PT1027:** Sweden. Jämtland, Aspås; 2011, *LH*; S: B184255; KJ657909, KJ657806, KJ658053. **PT1028:** Sweden. Gotland, Bunge; 2011, *LH*; S: B184252; KJ657895, KJ657792, KJ658039. **PT1031:** Sweden. Gotland, Rute; 2011, *LH*; S: B184251; KJ657896, KJ657793, KJ658040. **PT1033:** Sweden. Gotland, Othem; 2011, *LH*; S: B184249; KJ657897, KJ657794, KJ658041. **PT1037:** Sweden. Härjedalen, Storsjö; 1990, *N.Hakelier*; S: B39106; KJ657910, KJ657807, KJ658054. **PT1050:** Sweden. Öland, S. Möckleby; 1996, *LH & A.Kooijman*; S: B1121; KJ657887, KJ657783, KJ658031. **PT1051:** Sweden. Öland, Sandby; 2010, *LH*; S: B174622; KJ657888, KJ657784, KJ658032. **PT1052:** Sweden. Öland, Skogsby; 2010, *LH*; S: B174634; KJ657889, KJ657785, KJ658033. **PT1053:** Sweden. Öland, Sandby; 2010, *LH*; S: B174720; KJ657890, KJ657786, KJ658034. **PT1054:** Sweden. Gotland, Endre; 1989, *LH*; S: B38827; KJ657898, KJ657795, KJ658042. **PT1055:** Sweden. Gotland, Bro; 1989, *LH*; S: B38829; KJ657899, KJ657796, KJ658043. **PT1056:** Sweden. Gotland, Hejdeby; 1989, *LH*; S: B38830; KJ657900, KJ657797, KJ658044. **PT1057:** Sweden. Gotland, Boge; 1995, *LH*; S: B38832; KJ657901, KJ657798, KJ658045. **PT1058:** Sweden. Gotland, Fårö; 2003, *LH*; S: B84455; KJ657902, KJ657799, KJ658046. **PT1059:** Sweden. Västergötland, Österplana; 1961, *N.Hakelier*; S: B38903; KJ657903, KJ657800, KJ658047. **PT1060:** Sweden. Gästrikland, Orarna; 1967, *C.Rune & H.Sjörs*; S: B39109; KJ657907, KJ657804, KJ658051. **PT1061:** Sweden. Härjedalen, Storsjö; 2002, *LH*; S: B72102; KJ657911, KJ657808, KJ658055. **PT1062:** Sweden. Härjedalen, Storsjö; 2002, *LH*; S: B75428; KJ657912, KJ657809, KJ658056. **PT1063:** Sweden. Härjedalen, Tännäs; 2002, *LH*; S: B75521; KJ657913, KJ657810, KJ658057. **PT1064:** Sweden. Härjedalen, Ljusnedal; 2007, *LH*; S: B121997; KJ657914, KJ657811, KJ658058. **PT1065:** Sweden. Härjedalen, Storsjö; 2007, *LH*; S: B121998; KJ657915, KJ657812, KJ658059. **PT1066:** Sweden. Jämtland, Frostviken; 1997, *LH & A.Kooijman*; S: B1404; KJ657916, KJ657813, KJ658060. **PT1067:** Sweden. Jämtland, Frostviken; 2009, *LH*; S: B165509; KJ657917, KJ657814, KJ658061. **PT1068:** Sweden. Jämtland, Frostviken; 2009, *LH*; S: B165492; KJ657918, KJ657815, KJ658062. **PT1069:** Sweden. Jämtland, Frostviken; 2009, *LH*; S: B164581; KJ657919, KJ657816, KJ658063. **PT1070:** Sweden. Lycksele Lappmark, Tärna; 1965, *G.Een*; S: B39093; KJ657926, KJ657822, KJ658070. **PT1071:** Sweden. Lycksele Lappmark, Tärna; 2012, *LH et al.*; S: B195233; KJ657927, KJ657823, KJ658071. **PT1072:** Sweden. Pite Lappmark, Arjeplog; 1960, *G.Een*; S: B79200; KJ657928, KJ657824, KJ658072. **PT1073:** Sweden. Pite Lappmark, Arjeplog; 1960, *G.Een*; S: B88692; KJ657929, KJ657825, KJ658073. **PT1074:** Sweden. Lule Lappmark, Padjelanta; 1998, *T.-B.Engelmark*; S: B63794; KJ657941, KJ657837, KJ658085. **PT1075:** Sweden. Torne Lappmark, Jukkasjärvi; 1941, *W.R.Uggla*; S: B39185; KJ657942, KJ657838, KJ658086. **PT1076:** Sweden. Torne Lappmark, Jukkasjärvi; 2002, *LH*; S: B73404; KJ657943, KJ657839, KJ658087. **PT1077:** Sweden. Torne Lappmark, Jukkasjärvi; 2002, *LH*; S: B73440; KJ657944, KJ657840, KJ658088. **PT1078:** Sweden. Torne Lappmark, Karesuando; 1980, *LH*; S: B181705; KJ657945, KJ657841, KJ658089. **PT1079:** Norway. Troms, Bardu; 2008, *LH*; S: B138431; KJ657946, KJ657842, KJ658090. **PT1088:** Sweden. Västergötland, Österplana; 1980, *T.Hallingbäck 1787*; S: B195953; KJ657904, KJ657801, KJ658048. **PT1089:** Sweden. Lule Lappmark, Jokkmokk; 2005, *T.Hallingbäck 43350*; S: B195954; KJ657947, KJ657843, KJ658091. **PT1090:** Sweden. Lule Lappmark, Jokkmokk; 2002, *H.Weibull 38272*; S: B195955; KJ657948, KJ657844, KJ658092. **PT1091:** Sweden. Lule Lappmark, Jokkmokk; 1984, *T.Hallingbäck 1786*; S: B195956; KJ657949, KJ657845, KJ658093. **PT1092:** Norway. Sör-Tröndelag, Röros; 1966, *R.Elven*; TRH: 141234; KJ657920, KJ657817, KJ658064. **PT1093:** Norway. Sör-Tröndelag, Oppdal; 1978, *S.A.Hatlelid*; TRH: 141225; KJ657921, KJ657818, KJ658065. **PT1094:** Norway. Möre og Romsdal, Sunndal; 2000, *T.Prestö*; TRH: B-6970; KJ657908, KJ657805, KJ658052. **PT1096:** Norway. Nord-Tröndelag, Meråker; 1982, *A.A.Frisvoll*; TRH: 164961; KJ657923, KJ657819, KJ658067. **PT1097:** Norway. Troms, Storfjord. Skibotn; 2012, *K.Hassel & I.H.Kålås*; TRH: B-772272; KJ657950, KJ657846, KJ658094. **PT1121:** Norway. Nordland, Bodø; 2013, *LH*; S: B197288; KJ657930, KJ657826, KJ658074. **PT1115:** Sweden. Västergötland, Kinnekulle; 1938, *N.Albertson*; S: B38905; KJ657905, KJ657802, KJ658049. **PT1116:** Sweden. Västergötland, Kinnekulle; 1946, *P.O.Nyman*; S: B38920; KJ657906, KJ657803, KJ658050. **PT1117:** Sweden. Härjedalen, Tännäs; 1982, *LH*; S: B181704; KJ657924, KJ657820, KJ658068. **PT1118:** Sweden. Härjedalen, Tännäs; 1982, *LH*; S: B39108; KJ657925, KJ657821, KJ658069. **PT1121:** Norway. Nordland, Bodø; 2013, *LH*; S: B197288; KJ657930, KJ657826, KJ658074. **PT1122:** Norway. Nordland, Sørfold; 2013, *LH*; S: B197292; KJ657931, KJ657827, KJ658075. **PT1123:** Norway. Nordland, Sørfold; 2013, *LH*; S: B197293; KJ657932, KJ657828, KJ658076. **PT1124:** Norway. Nordland, Sørfold; 2013, *LH*; S: B197295; KJ657933, KJ657829, KJ658077. **PT1125:** Norway. Nordland, Saltdal; 2013, *LH*; S: B197297; KJ657934, KJ657830, KJ658078. **PT1126:** Norway. Nordland, Saltdal; 2013, *LH*; S: B197300; KJ657935, KJ657831, KJ658079. **PT1127:** Norway. Nordland, Saltdal; 2013, *LH*; S: B197301; KJ657936, KJ657832, KJ658080. **PT1128:** Norway. Nordland, Saltdal; 2013, *LH*; S: B197302; KJ657937, KJ657833, KJ658081. **PT1129:** Norway. Nordland, Fauske; 2013, *LH*; S: B197303; KJ657938, KJ657834, KJ658082. **PT1130:** Norway. Nordland, Fauske; 2013, *LH*; S: B197305; KJ657939, KJ657835, KJ658083. **PT1203:** Sweden. Gotland, Gammelgarn; 2016, *LH*; S: B237637; OP820059, OP831241, OP831226. **PT1208:** Sweden. Gotland, Fleringe, Tvärlingsmyr; 2013, *LH & I.Bisang*; S: B196901; OP820060, OP831242, OP831227. **PT1239:** Norway. Nordland, Bodø, Oddan; 2014, *LH*; S: B206973; OP820061, OP831243, OP831228. **PT1247:** Sweden. Pite Lappmark, Arjeplog, Mávasjávrre; 2015, *LH et al.*; S: B226803; OP820062, OP831244, OP831229. **PT1259:** Sweden. Öland, Kastlösa, Tingstad flisor; 2014, *LH*; S: B210745; OP820063, OP831245, OP831230. **PT1273:** Sweden. Härjedalen, Storsjö, Mt. Veaketjahke; 2014, *LH*; S: B208671; OP820064, OP831246, OP831231. **PT1278:** Sweden. Härjedalen, Tännäs, Mt. Giertebåvne; 2014, *LH*; S: B208672; OP820065, OP831247, OP831232. **PT1281:** Sweden. Härjedalen, Tännäs, Lake Kläpptjärnen; 2014, *LH*; S: B208665; OP820066, OP831248, OP831233. **PT1282:** Sweden. Västergötland, Österplana; 2018, *LH*; S: B279630; OP820067, OP831249, OP831234. **PT1283:** Sweden. Västergötland, Österplana, Axvall farm; 2018, *LH*; S: B279631; OP820068, OP831250, OP831235. **PT1284:** Sweden. Västergötland, Österplana; 2018, *LH*; S: B279632; OP820069, OP831251, OP831236. **PT1287:** Sweden. Öland, Kastlösa, St. Dalby-Bårby; 2014, *LH*; S: B210728; OP820070, OP831252, OP831237. **PT1293:** Sweden. Öland, Resmo; 2010, *LH*; S: B174724; OP820071, OP831253, OP831238. **PT1296:** Sweden. Gotland, Östergarn, Svartdal; 2019, *LH*; S: B290225; OP820072, OP831254, OP831239. **PT1301:** Sweden. Gotland, Öja, Unghanse; 2019, *LH & I.Bisang*; S: B290215; OP820073, OP831255, OP831240.

**B**

**PT1200_F:** Sweden. Gotland, Etelhem, Branden; 2016, *LH*; S: B238800. **PT1201_F:** Sweden. Gotland, Othem, Filehajdar; 2015, *LH*; S: B220780. **PT1202_F:** Sweden. Gotland, Östergarn, Falhammars; 2016, *LH*; S: B236512. **PT1203 (PT1203)_M:** Sweden. Gotland, Gammelgarn; 2016, *LH*; S: B237637. **PT1204_F:** Sweden. Gotland, Hejnum, Hejnum hällar; 2015, *LH*; S: B222087. **PT1205_F:** Sweden. Gotland, Bäl, Bälsalver; 2015, *LH*; S: B222085. **PT1206_F:** Sweden. Gotland, Fleringe, Gajsthajd; 2014, *LH et al.*; S: B205551. **PT1207_F:** Sweden. Gotland, Hellvi, Kyllajhajdar; 2013, *LH*; S: B196913. **PT1208 (PT1208)_M:** Sweden. Gotland, Fleringe, Tvärlingsmyr; 2013, *LH & I.Bisang*; S: B196901. **PT1209_F:** Sweden. Gotland, Hangvar, Klockaremyr; 2013, *LH*; S: B196936. **PT1210 (PT1055)_F:** Sweden. Gotland, Bro; 1989, *LH G89-230*; S: B38829. **PT1211 (PT1054)_F:** Sweden. Gotland, Endre; 1989, *LH G89-42*; S: B38827. **PT1212 (PT1057)_F:** Sweden. Gotland, Boge; 1995, *LH*; S: B38832. **PT1213_F:** Sweden. Gotland, Fårö, Hammars; 1995, *LH*; S: B38831. **PT1214_F:** Sweden. Gotland, Stenkyrka, Bromyr; 2001, *LH*; S: B62359. **PT1216_F:** Sweden. Gästrikland, Gävle kn., Orarna; 2009, *T.Troschke*; S: B169631. **PT1217 (PT1066)_F:** Sweden. Jämtland, Frostviken; 1997, *LH & A.Kooijman*; S: B1404. **PT1218 (PT1067)_M:** Sweden. Jämtland, Frostviken; 2009, *LH*; S: B165509. **PT1219 (PT1068)_M:** Sweden. Jämtland, Frostviken; 2009, *LH*; S: B165492. **PT1220 (PT1069)_M:** Sweden. Jämtland, Frostviken; 2009, *LH*; S: B164581. **PT1221 (PT1074)_F:** Sweden. Lule Lappmark, Padjelanta; 1998, *T.-B.Engelmark*; S: B63794. **PT1222 (PT1089)_F:** Sweden. Lule Lappmark, Jokkmokk; 2005, *T.Hallingbäck*; S: B195954. **PT1223_F:** Sweden. Torne Lappmark, Jukkasjärvi, Kopparåsen; 2003, *T.Hallingbäck*; S: B217264. **PT1224 (PT1076)_F:** Sweden. Torne Lappmark, Jukkasjärvi; 2002, *LH*; S: B73404. **PT1225 (PT1121)_F:** Norway. Nordland, Bodø; 2013, *LH*; S: B197288. **PT1226 (PT1130)_F:** Norway. Nordland, Fauske; 2013, *LH*; S: B197305. **PT1227 (PT1129)_F:** Norway. Nordland, Fauske; 2013, *LH*; S: B197303. **PT1228 (PT1128)_M:** Norway. Nordland, Saltdal; 2013, *LH*; S: B197302. **PT1229 (PT1127)_F:** Norway. Nordland, Saltdal; 2013, *LH*; S: B197301. **PT1230 (PT1126)_F:** Norway. Nordland, Saltdal; 2013, *LH*; S: B197300. **PT1231 (PT1123)_F:** Norway. Nordland, Sørfold; 2013, *LH*; S: B197293. **PT1232 (PT1122)_F:** Norway. Nordland, Sørfold; 2013, *LH*; S: B197292. **PT1233_F:** Norway. Nordland, Bodø, Skutvikodden-Skålbunesodden; 2014, *LH*; S: B206984. **PT1235_F:** Norway. Nordland, Bodø, Knaplundsøya; 2014, *LH*; S: B206996. **PT1236_F:** Norway. Nordland, Bodø, Knaplundsøya; 2014, *LH*; S: B207003. **PT1237_F:** Norway. Nordland, Bodø, Mørkved; 2014, *LH*; S: B206953. **PT1238_F:** Norway. Nordland, Bodø, Mørkved; 2014, *LH*; S: B206957. **PT1239 (PT1239)_M:** Norway. Nordland, Bodø, Oddan; 2014, *LH*; S: B206973. **PT1240_F:** Norway. Nordland, Bodø, Knaplundsøya; 2014, *LH*; S: B207007. **PT1241_F:** Norway. Nordland, Fauske, Øyneshögda; 2014, *LH & I.Bisang*; S: B207041. **PT1242_F:** Norway. Nordland, Fauske, Øyneshögda; 2014, *LH & I.Bisang*; S: B207043. **PT1243_F:** Norway. Nordland, Fauske, Stranda; 2014, *LH & I.Bisang*; S: B207009. **PT1244_F:** Norway. Nordland, Fauske, Stranda; 2014, *LH & I.Bisang*; S: B207018. **PT1245_F:** Sweden. Pite Lappmark, Arjeplog, Mávasjávrre; 2015, *LH et al.*; S: B226804. **PT1246_F:** Sweden. Pite Lappmark, Arjeplog, Mávasjávrre; 2015, *LH et al.*; S: B226160. **PT1247 (PT1247)_M:** Sweden. Pite Lappmark, Arjeplog, Mávasjávrre; 2015, *LH et al.*; S: B226803. **PT1248_F:** Sweden. Pite Lappmark, Arjeplog, Mávasjávrre; 2015, *LH et al.*; S: B227607. **PT1249_F:** Sweden. Pite Lappmark, Arjeplog, Mávasjávrre; 2015, *LH et al.*; S: B226159. **PT1250_F:** Sweden. Pite Lappmark, Arjeplog, Mávasjávrre; 2015, *LH et al.*; S: B226158. **PT1251_F:** Sweden. Öland, Resmo, Lake Möckelmossen; 1996, *LH & A.Kooijman*; S: B1123. **PT1252 (PT1050)_F:** Sweden. Öland, S. Möckleby; 1996, *LH & A.Kooijman*; S: B1121. **PT1253_F:** Sweden. Öland, Sandby, S. Sandby; 2010, *LH*; S: B174632. **PT1255 (PT1051)_F:** Sweden. Öland, Sandby; 2010, *LH*; S: B174622. **PT1256 (PT1052)_M:** Sweden. Öland, Skogsby; 2010, *LH*; S: B174634. **PT1257_F:** Sweden. Öland, Stenåsa, Ebbelunda; 2010, *LH*; S: B174618. **PT1258_F:** Sweden. Öland, alvar SE of Eriksöre; 2010, *LH*; S: B174626. **PT1259 (PT1259)_M:** Sweden. Öland, Kastlösa, Tingstad flisor; 2014, *LH*; S: B210745. **PT1260_F:** Sweden. Öland, Mörbylånga, Bårby; 2014, *LH*; S: B210725. **PT1261_F:** Sweden. Öland, Torslunda, Lenstad; 2014, *LH*; S: B210720. **PT1262_F:** Sweden. Öland, Resmo; 2015, *LH & I.Bisang*; S: B222103. **PT1263_F:** Sweden. Öland, Räpplinge, Greby; 2016, *LH*; S: B236800. **PT1264_F:** Sweden. Öland, Persnäs, Sandvik; 2016, *LH*; S: B236799. **PT1265_F:** Sweden. Öland, Räpplinge, Greby; 2016, *LH*; S: B237017. **PT1266_F:** Sweden. Öland, Ventlinge; 2016, *LH*; S: B236797. **PT1267 (PT1062)_F:** Sweden. Härjedalen, Storsjö; 2002, *LH*; S: B75428. **PT1268 (PT1061)_F:** Sweden. Härjedalen, Storsjö; 2002, *LH*; S: B72102. **PT1269 (PT1063)_F:** Sweden. Härjedalen, Tännäs; 2002, *LH*; S: B75521. **PT1270 (PT1064)_F:** Sweden. Härjedalen, Ljusnedal; 2007, *LH*; S: B121997. **PT1271_F:** Sweden. Härjedalen, Storsjö, Svaaletjahkh; 2014, *LH*; S: B208666. **PT1272_F:** Sweden. Härjedalen, Storsjö, Svaaletjahkh; 2014, *LH*; S: B208669. **PT1273 (PT1273)_M:** Sweden. Härjedalen, Storsjö, Mt. Veaketjahke; 2014, *LH*; S: B208671. **PT1274_F:** Sweden. Härjedalen, Tännäs, Kliehpie-Joltere; 2014, *LH*; S: B208653. **PT1275_F:** Sweden. Härjedalen, Tännäs, Mt. Kliehpie; 2014, *LH*; S: B208657. **PT1276_F:** Sweden. Härjedalen, Tännäs, Mt. Kliehpie; 2014, *LH*; S: B208658. **PT1277_F:** Sweden. Härjedalen, Tännäs, Lake Kläpptjärnen; 2014, *LH*; S: B208664. **PT1278 (PT1278)_M:** Sweden. Härjedalen, Tännäs, Mt. Giertebåvne; 2014, *LH*; S: B208672. **PT1279_F:** Sweden. Härjedalen, Tännäs, Mt. Joltere; 2014, *LH*; S: B208656. **PT1280_F:** Sweden. Härjedalen, Tännäs, Mt. Kliehpie; 2014, *LH*; S: B208660. **PT1281 (PT1281)_M:** Sweden. Härjedalen, Tännäs, Lake Kläpptjärnen; 2014, *LH*; S: B208665. **PT1282 (PT1282)_M:** Sweden. Västergötland, Österplana; 2018, *LH*; S: B279630. **PT1283 (PT1283)_M:** Sweden. Västergötland, Österplana Axvall farm; 2018, *LH*; S: B279631. **PT1284 (PT1284)_M:** Sweden. Västergötland, Österplana; 2018, *LH*; S: B279632. **PT1285_F:** Sweden. Öland, Ventlinge, Lunda; 2016, *LH*; S: B236796. **PT1286_F:** Sweden. Öland, Stenåsa, Ebbelunda; 2010, *LH*; S: B174620. **PT1287 (PT1287)_M:** Sweden. Öland, Kastlösa, St. Dalby-Bårby; 2014, *LH*; S: B210728. **PT1288_F:** Sweden. Öland, Kastlösa, St. Dalby; 2014, *LH*; S: B210732. **PT1289_F:** Sweden. Öland, Kastlösa, St. Dalby; 2014, *LH*; S: B210731. **PT1290_F:** Sweden. Öland, Kastlösa, St. Dalby-Tingstad flisor; 2014, *LH*; S: B210743. **PT1291_F:** Sweden. Öland, Mörbylånga, Bårby; 2014, *LH*; S: B210726. **PT1292_F:** Sweden. Öland, Alböke; 2016, *LH*; S: B237274. **PT1293 (PT1293)_M:** Sweden. Öland, Resmo; 2010, *LH*; S: B174724. **PT1294_M:** Sweden. Öland, Eriksöre; 2010, *LH*; S: B174795. **PT1295_F:** Sweden. Gotland, Buttle, Hägsarve; 2016, *LH*; S: B236511. **PT1296 (PT1296)_M:** Sweden. Gotland, Östergarn, Svartdal; 2019, *LH*; S: B290225. **PT1297_F:** Sweden. Gotland, Kräklingbo, Hajdeby; 2019, *LH*; S: B290227. **PT1298_F:** Sweden. Gotland, Kräklingbo, Trosingsgärdet; 2019, *LH*; S: B290228. **PT1299_F:** Sweden. Gotland, Öja, Unghanse; 2019, *LH & I.Bisang*; S: B290212. **PT1300A_F:** Sweden. Gotland, Öja, Unghanse; 2019, *LH & I.Bisang*; S: B290213. **PT1301A (PT1301A)_M:** Sweden. Gotland, Öja, Unghanse; 2019, *LH & I.Bisang*; S: B290215. **PT1302A_F:** Sweden. Gotland, Hejde, Hajdskogen; 2019, *LH*; S: B290216. **PT1303A_F:** Sweden. Gotland, Othem, Filehajdar; 2019, *LH & I.Bisang*; S: B290219. **PT1304A_F:** Sweden. Gotland, Othem, Filehajdar; 2019, *LH & I.Bisang*; S: B290222.
