# Supplementary material for: Life-history characteristics and historical factors are important to explain regional variation in reproductive traits and genetic diversity in perennial mosses
Source: Ann Bot. 2023 Mar 16;132(1):29–42. doi: 10.1093/aob/mcad045 (PMC10550275; doi:10.1093/aob/mcad045)
Supplement: mcad045_suppl_Supplementary_Data [file mcad045_suppl_supplementary_data.docx]

Supplementary DATA

File name: *Reproduction_variation supplementary_Table S1.docx*

Bryophyte life cycle and bryophyte specific terminology

File name: *Reproduction_variation supplementary_Table S2.xlsx*

Studied material of *Drepanocladus trifarius* and *D. turgescens* for the analyses of reproductive traits and intraspecific genetic variation, including the indication of the different subsets that were used for different analyses.

Study region; Data sets; Locality (Locality country: SE, Sweden; NO, Norway; Geographic locality); Longitude °E; Latitude °N; Collector; Collection date; Collection institution (Herbarium); Registration number in the respective herbarium; Reproductive state, SPOR: samples bearing sporophytes, R: samples bearing male or female reproductive structures but no sporophytes, NR: samples without reproductive structures; Sex, M: male, F: female, F/M samples containing both F and M; Sex identified based on reproductive organs in R-samples or by molecular methods in NR-samples, n/a: sex in the sample not identified. DNA sample_no is the respective sample identifier for DNA extractions.

*Drepanocladus trifarius*: Study regions, north: northern Sweden, south-central: southern and central Sweden. Data set R: reproductive samples (N=84, 4 samples with both sexes) for phenotypic sex ratio assessment. Dataset GenVar: Samples analysed to assess genetic diversity from north (42) and south-central (44) Sweden. Dataset GenVar: Samples and their sex analysed to assess genetic diversity between female (41) and male (45) samples. ‘DNA sample no_genvar’ is the respective sample identifier for DNA extractions.

*Drepanocladus turgescens*: Study regions, BA: Baltic Sea islands, MA: mainland of Sweden and Norway. Data set R: reproductive samples (101, 5 samples with both sexes), Data set NR: non-reproductive samples (102), for sex ratio assessments. Data set GenVar: Samples analysed to assess genetic diversity in BA (33) and MA (55). ‘DNA sample no_genvar’ is the respective sample identifier. Data set GenSex: Samples and their sex (identified based on reproductive organs in R and ‘spor’-samples, or by molecular methods in NR-samples) analysed to compare genetic diversity in female and male samples in BA (31) and MA (37). ‘DNA sample no_NR’ is the sample identifier to identify sex in non-reproductive samples.

See Material and Methods for further details on the sample design.

File name: *Reproduction_variation supplementary_Table S3.docx*

A. Sample localities, associated data and GenBank accession numbers for *Drepanocladus trifarius* and *D. turgescens* studied for genetic variation. B. Samples used for molecular sex identification in *D. turgescens*.

File name: *Reproduction_variation supplementary_Figs S1_S2.docx*

Sample locations of *Drepanocladus trifarius* (Fig. S1) and *Drepanocladus turgescens* (Fig. S2) in Scandinavia. Dots on maps at the presented scale may represent several occurrences of the species, especially in *D. turgescens* that is more irregularly distributed across Scandinavia than *D. trifarius* due to habitat restrictions. Different subsets were used for the different analyses; see Material and Methods and Supplementary Material, Table S2, for details on the sampling protocols.

**Fig. S1. A** Location of *D. trifarius* samples for reproductive trait analyses. Reproductive F, samples bearing female reproductive structures but no sporophytes. Reproductive M, samples bearing male reproductive structures. Reproductive F_M, samples bearing female (not sporophytes) and male reproductive structures. SPOR, samples bearing sporophytes (N=10). NR, samples without reproductive structures. **Fig. S1. B** Location of *D. trifarius* samples for genetic analyses. GenVAR, samples out of total data set in Fig. S1A analysed for genetic variation; GenVAR_ADD, 19 samples from Hedenäs (2019) added to GenVAR.

**Fig. S2. A** Location of *D. turgescens* samples for reproductive trait analyses. Sampling intensity per unit area was higher on the Baltic Sea islands than on the mainland to reflect occurrence density. Reproductive F, samples bearing female reproductive structures but no sporophytes. Reproductive M, samples bearing male reproductive structures. Reproductive F_M, samples bearing female (not sporophytes) and male reproductive structures. SPOR: samples bearing sporophytes (N=24). NR, samples without reproductive structures. **Fig. S2. B.** Location of the non-reproductive subset of *D. turgescens* samples analysed in this study. F, female samples. M, male samples. **Fig. S2. C** Location of *D. turgescens* samples for genetic analyses. GenVAR, samples out of total data set in Fig. S2A analysed for genetic variation. GenVAR_ADD, ten samples from Hedenäs (2014) added to GenVAR.
